# Catalysis by Bidentate Iodine(III)-Based Halogen Donors: Surpassing the Activity of Strong Lewis Acids

**DOI:** 10.1021/acs.joc.1c00534

**Published:** 2021-03-25

**Authors:** Susana Portela, Jorge J. Cabrera-Trujillo, Israel Fernández

**Affiliations:** Departamento de Química Orgánica I and Centro de Innovación en Química Avanzada (ORFEO-CINQA), Facultad de Ciencias Químicas, Universidad Complutense de Madrid, 28040 Madrid, Spain

## Abstract

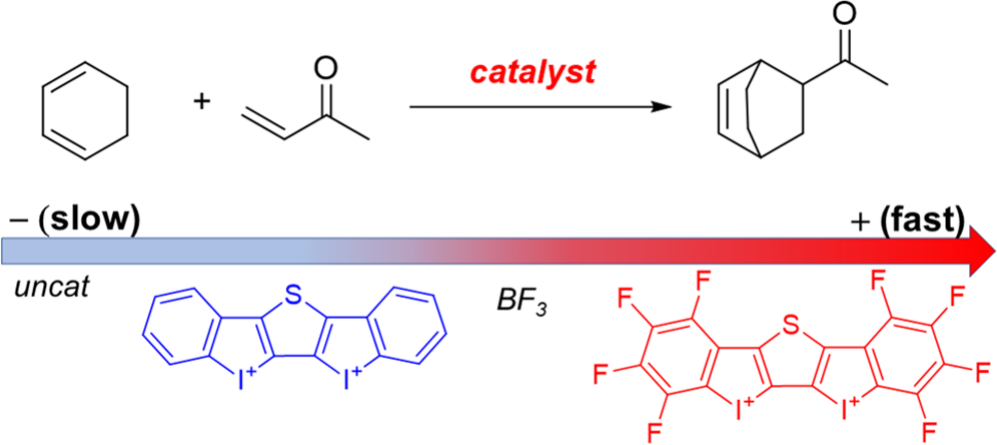

The poorly understood mode of activation and catalysis of bidentate iodine(III)-based halogen donors have been quantitatively explored in detail by means of state-of-the-art computational methods. To this end, the uncatalyzed Diels–Alder cycloaddition reaction between cyclohexadiene and methyl vinyl ketone is compared to the analogous process mediated by a bidentate iodine(III)-organocatalyst and by related, highly active iodine(I) species. It is found that the bidentate iodine(III)-catalyst accelerates the cycloaddition by lowering the reaction barrier up to 10 kcal mol^–1^ compared to the parent uncatalyzed reaction. Our quantitative analyses reveal that the origin of the catalysis is found in a significant reduction of the steric (Pauli) repulsion between the diene and dienophile, which originates from both a more asynchronous reaction mode and a significant polarization of the π-system of the dienophile away from the incoming diene. Notably, the activity of the iodine(III)-catalyst can be further enhanced by increasing the electrophilic nature of the system. Thus, novel systems are designed whose activity actually surpasses that of strong Lewis acids such as BF_3_.

## Introduction

Noncovalent interactions arguably play a key role in catalysis.^1^ Indeed, these relatively weak interactions have been invoked to control, to a considerable extent, not only the reactivity but also the selectivity (from regio- or chemoselectivity to enantioselectivity) of different catalyzed transformations ranging from organocatalysis to transition-metal-mediated process.^1^ In particular, halogen bonding (i.e., the interaction involving an electrophilic halogen substituent and a Lewis base)^2,3^ has been established in organocatalysis in the last decade and successfully applied to a number of organic reactions.^4^ So far, most of these organocatalysts are typically based on iodine(I) derivatives, either cationic or neutral species (the former being usually more active than the latter).^4,5^

**Scheme 1:**
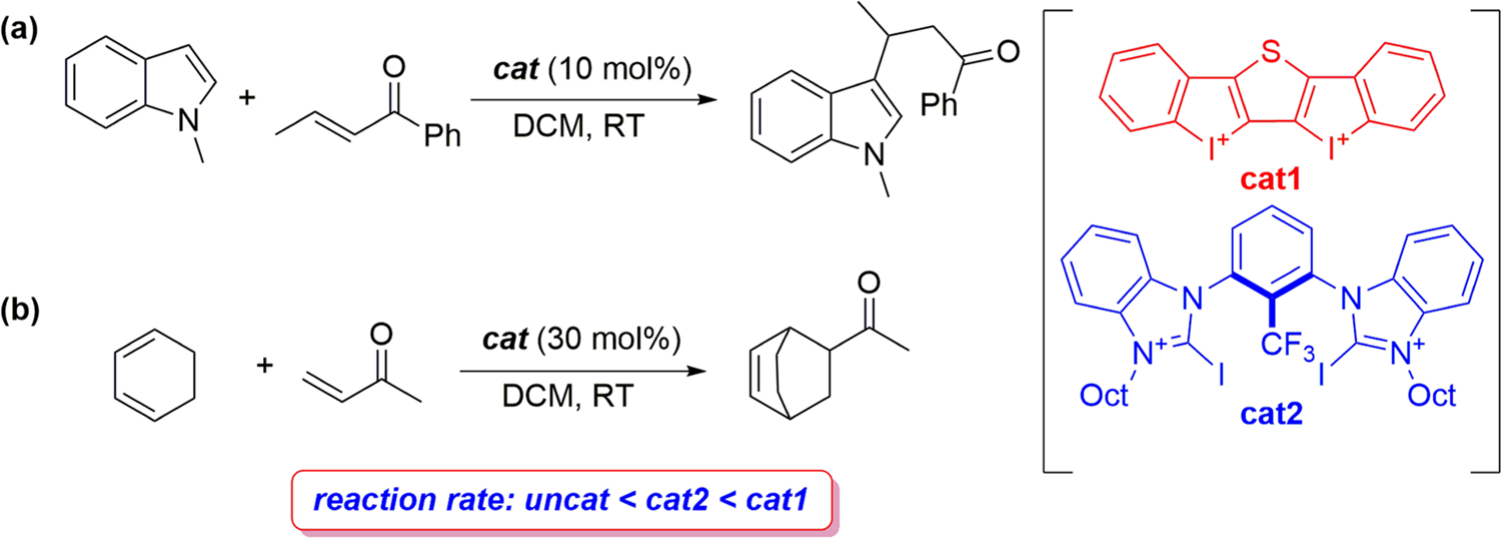
Michael Addition Reaction (a) and Diels–Alder Cycloaddition (b) Mediated by the Iodine(III)-Halogen Donor **cat1** Reported by Huber and Co-Workers (See ref (11))

In contrast, iodine(III)-based halogen-donor catalysts are comparatively much more underdeveloped. In this regard, the studies by Han and Liu,^6^ Huber,^7^ Aoshima,^8^ and Nachtsheim^9^ using iodonium salts should be particularly highlighted. Interestingly, Huber and co-workers very recently reported that the bidentate bis(iodolium) salt **cat1**, initially prepared by Wu and Yoshikai,^10^ is able to catalyze fundamental processes in organic chemistry such as Michael addition and Diels–Alder cycloaddition reactions (Scheme 1).^11^ The catalytic activity of this species, which in the authors’ own words, “...approach the potency of Lewis acids like BF_3_”,^11^ is proven to outperform that of the currently strongest iodine(I)-based organocatalyst **cat2**. For instance, while only 41% of the Michael addition product was formed after 1 h when using **cat2**, a 74% of the corresponding Michael adduct was produced in the same reaction time when using **cat1** (Scheme 1a). The enhanced catalytic activity of this species is mainly ascribed to the bidentate nature of the organocatalyst–substrate binding, which preorganizes and activates the α,β-unsaturated ketone. Despite that, very little is known about the ultimate factors responsible for the remarkable acceleration induced by **cat1**, which hampers the future development of novel, highly active species.

On the other hand, we recently found, using state-of-the-art computational methods, that not only strong Lewis acids (such as AlCl_3_ or BF_3_)^12^ but other catalysts able to establish noncovalent interactions with the substrate, including hydrogen and halogen bonds,^13^ accelerate Michael addition and Diels–Alder reactions by reducing the Pauli repulsion between the key π-orbitals of the reactants involved in the transformation.^14^ This so-called “Pauli-repulsion lowering” concept challenges the textbook “LUMO-lowering” concept,^15^ widely used to rationalize the mode of activation of these catalysts. In this sense, the catalysis by the bidentate iodine(III)-derivative **cat1** reported by Huber and co-workers^11^ represents a paramount opportunity to apply our methodology toward a quantitative understanding of the actual reasons behind the enhanced activity of this bidentate organocatalyst. The insight gained in this study will be then used to rationally design new halogen-donor systems, which, as described below, not only approach but even surpass the catalytic activity of strong Lewis acids such as BF_3_.

## Results and Discussion

**Figure 1:**
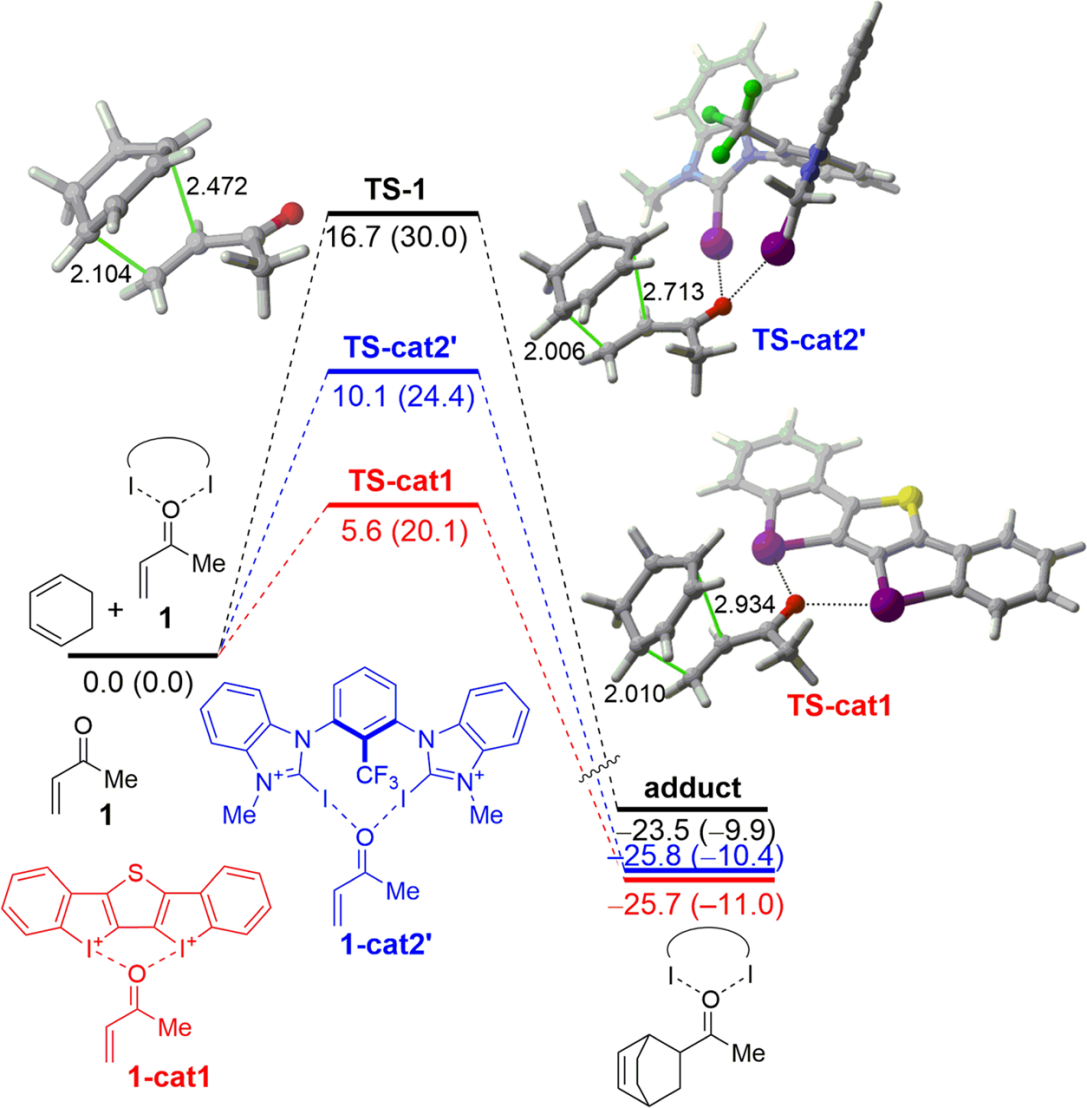
Computed reaction profiles for the Diels–Alder cycloaddition reactions between cyclohexadiene and MVK **1** (black), **1-cat1** (red), and **1-cat2′** (blue). Relative energies (free energies, within parentheses) and bond distances are given in kcal/mol and angstroms, respectively. All data have been computed at the PCM(DCM)-B3LYP-D3/def2-TZVPP//PCM(DCM)-B3LYP-D3/def2-SVP level.

We focused on the experimentally described Diels–Alder cycloaddition reaction involving methyl vinyl ketone (MVK, **1**) and cyclohexadiene (Scheme 1b). We exclusively focused on the endo-approach as it is kinetically preferred (ΔΔ*G*^≠^ = 2.6 kcal/mol) over the corresponding exo-approach. The parent uncatalyzed process is compared to the analogous cycloaddition mediated by the bidentate iodine(III)-organocatalyst **cat1** (i.e., involving the **1-cat1** complex). For completeness, we also considered the process catalyzed by the iodine(I)-derivative **cat2′**, a model catalyst of **cat2** where the octyl substituents were replaced by methyl groups. Our calculations (Figure 1) indicate that in all cases, the cycloaddition proceeds in a concerted yet asynchronous manner through the corresponding six-membered transition state (TS), leading to the exergonic formation (Δ*G*_R_ ∼ −10 kcal/mol) of the corresponding cycloadduct. From the data in Figure 1, it becomes evident that the **cat1**-catalyzed cycloaddition requires a much lower activation than the parent uncatalyzed process (ΔΔ*G*^≠^ = 9.9 kcal/mol). The situation involving iodine(I)-organocatalyst **cat2′** is intermediate between the uncatalyzed and **cat1**-catalyzed cycloadditions, which is consistent with the experimental and computational results (M06-2X/def2-TZVP(D) level) carried out by Huber and co-workers.^11,16^

**Figure 2:**
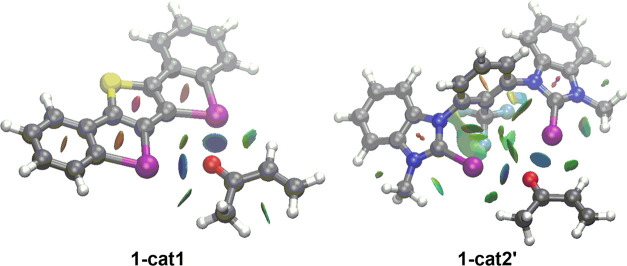
Contour plots of the reduced density gradient isosurfaces (density cutoff = 0.045 au) for the **1-cat1** and **1-cat2′** complexes. The greenish surfaces indicate attractive noncovalent interactions.

To understand the enhanced reactivity of the **cat1**-mediated reaction over the analogous uncatalyzed and **cat2′**-catalyzed processes, we first explored the bonding situation in the corresponding MVK-complexes **1-cat1** and **1-cat2′**. In both cases, the halogen bond donor catalyst forms a bidentate complex via a bifurcated halogen bond to MVK. This stabilizing double halogen bond interaction can be easily visualized by means of the NCIPLOT method.^17^ As shown in Figure 2, in both cases, there exist two clear noncovalent attractive interactions (greenish surfaces) between both iodine atoms of the catalyst and the carbonyl oxygen atom of the MVK, which confirms the occurrence of both halogen bonds. In addition, in both complexes, there are two additional stabilizing C–H···I interactions, which reflect the strong acceptor ability of the iodine atoms in both catalysts. In addition, the QTAIM (atom in molecules)^18^ method locates bond critical points (BCPs) between the carbonyl oxygen and iodine atoms and bond paths (BPs) running between them for both complexes. Interestingly, the computed positive values of the Laplacian of electron density (∇^2^ρ(*r*_c_) = +0.081 and +0.062, for **1-cat1** and **1-cat2′**, respectively) at the BCPs indicate that charge is locally depleted and, then, is consistent with the noncovalent nature of these C=O···I interactions.

Further quantitative analysis of the MVK-catalyst interaction can be gained with the help of the energy decomposition analysis (EDA) method.^19^ As shown in Table 1, the interaction between the Lewis base (i.e., the carbonyl group of MVK) and **cat1** is significantly higher than that involving **cat2′**, which confirms the higher electrophilic nature of the iodine(III)-catalyst. In both cases, the electrostatic interactions are nearly twice as strong as the orbital interactions, which agrees with the reported electrostatic nature of the halogen bonding.^2,3^ Despite that, both attractive interactions are comparatively much stronger in the **1-cat1** complex, which is translated into the computed stronger interaction. Thus, the computed trend in the interaction (Δ*E*_int_) between the dienophile and the catalyst as well as their main attractive contributions (Δ*V*_esltat_ and Δ*E*_orb_) follow the same trend as their relative activity (**cat1** > **cat2′**). Table 1 also shows the energy of the π*-molecular orbital of these dienophiles (i.e., located at the reactive C=C bond), which is the key in the cycloaddition reaction. As expected, the binding of the carbonyl group to the halogen-donor catalyst stabilizes this molecular orbital as compared to the parent MVK (ε_π*_ = −1.7 eV). This stabilization is higher in **1-cat** than in **1-cat2′**, which results in a lower (i.e., more favorable) HOMO(diene)–LUMO(dienophile) gap. Therefore, the traditional, textbook LUMO-lowering effect seems to be useful to rationalize the relative activity of these catalysts. We will show next, however, that the stabilization of the lowest unoccupied molecular orbital (LUMO) is not the actual factor controlling the catalysis by these halogen-donor organocatalysts.

**Table 1:** Energy Decomposition Analysis (in kcal/mol, ZORA-B3LYP-D3/TZ2P//PCM(DCM)-B3LYP-D3/def2-SVP Level) of the Interaction between MVK (**1**) and the Catalysts in the Corresponding **1-cat1** and **1-cat2′** Complexes

compound	**1-cat1**	**1-cat2′**
Δ*E*_int_	–34.8	–25.4
Δ*E*_Pauli_	39.1	23.5
Δ*V*_elstast_	–41.0	–27.8
Δ*E*_orb_	–27.4	–16.8
Δ*E*_disp_	–5.5	–4.4
ε_π*_ (eV)	–3.6	–2.9

**Figure 3:**
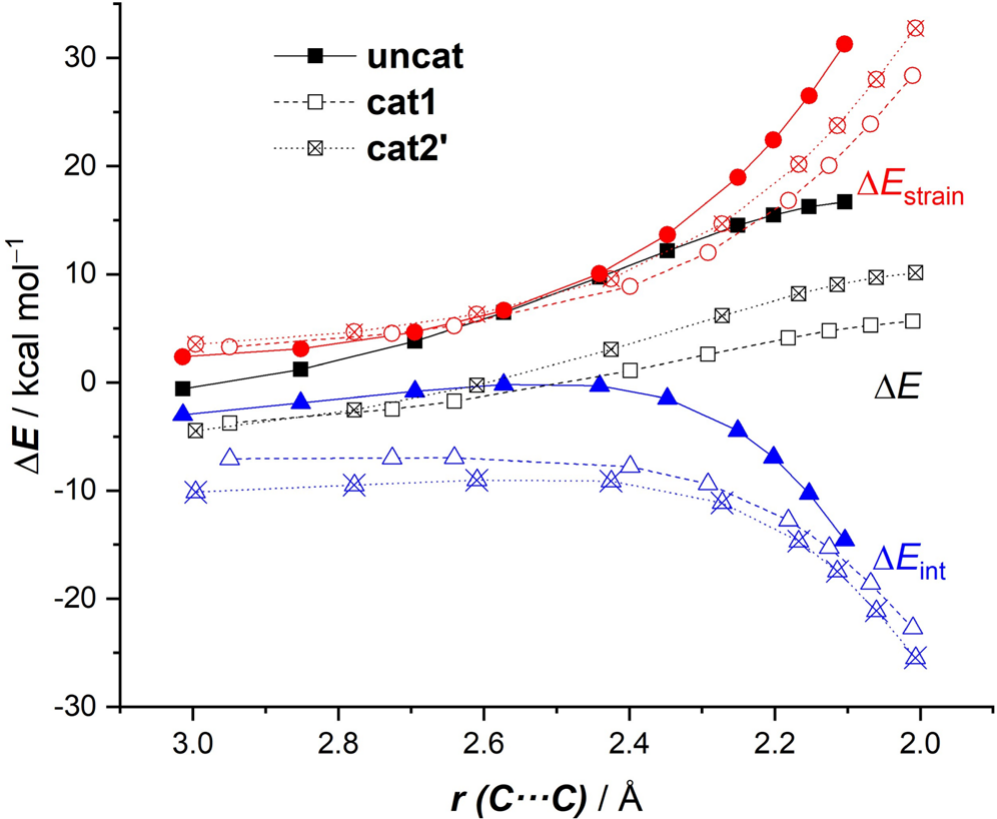
Comparative activation strain analyses of the Diels–Alder reactions between cyclohexadiene and uncoordinated (**none**) as well as **cat1**- and **cat2′**-bonded methyl vinyl ketone complexes projected onto the shorter C···C bond-forming distance. All data have been computed at the PCM(DCM)-B3LYP-D3/def2-TZVPP//PCM(DCM)-B3LYP-D3/def2-SVP level.

The activation strain model (ASM)^20^ approach was applied next to quantitatively understand the ultimate physical factors leading to the enhanced activity of the halogen-donor catalyst **cat1**. Figure 3 shows the corresponding activation strain diagrams (ASDs) for the uncatalyzed (**none**), **cat1-**, and **cat2′**-catalyzed cycloadditions along the reaction coordinate from the initial stages of the process up to the respective transition states and projected onto the shorter newly formed C···C bond between cyclohexadiene and the dienophile. This critical reaction coordinate undergoes a well-defined change throughout the reaction and has successfully been used in the past for the analysis of other cycloaddition reactions.^21^ It is found that the lower barrier computed for the **cat1**-mediated cycloaddition originates mainly from a combination of a much stronger interaction between the deformed reactants and less destabilizing strain energy along the entire transformation. Once again, the situation of the process mediated by the iodine(I)-catalyst **cat2′** is intermediate between the uncatalyzed and **cat1**-catalyzed reactions, not because of the interaction term (which is rather similar to that of the **cat1**-process) but to the strain term. The computed trend in Δ*E*_strain_ (**cat1** < **cat2′** < **none**) can be directly ascribed to the extent of the asynchronicity of the cycloaddition (**none**: Δ*r*_C···C_^TS^ = 0.37 Å < **cat2′**: Δ*r*_C···C_^TS^ = 0.71 Å < **cat1**: Δ*r*_C···C_^TS^ = 0.92 Å, where Δ*r*_C···C_^TS^ is the difference between the newly formed C···C bond lengths in the TS, see Figure 1), which leads to a lower degree of deformation of the diene since the C–C_β_ bond forms ahead of the C–C_α_ bond (for a plot of the variation of the strain associated with the deformation of the diene and dienophile along the reaction coordinate, see Figure S1).

**Figure 4:**
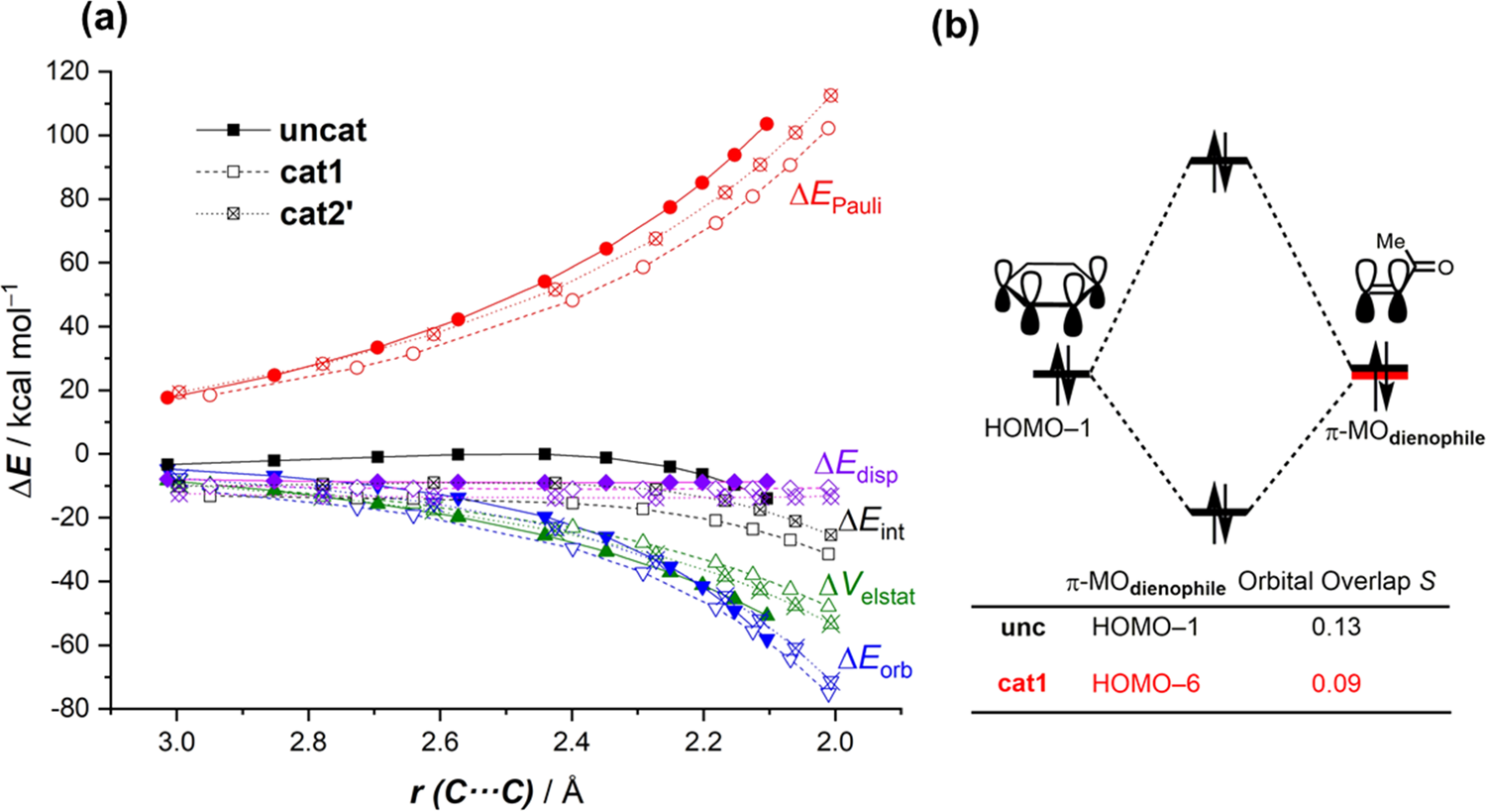
(a) Comparative energy decomposition analyses of the Diels–Alder reactions between cyclohexadiene and uncoordinated (**none**) as well as **cat1**- and **cat2′**-bonded methyl vinyl ketone complexes projected onto the shorter C···C bond-forming distance. (b) Molecular orbital diagram and the most significant occupied orbital overlaps of the cycloadditions. All data have been computed at the ZORA-B3LYP-D3/TZ2P//PCM(DCM)-B3LYP-D3/def2-SVP level.

The origin of the stronger interaction between the reactants computed for the catalyzed cycloadditions can be further quantitatively understood by using the energy decomposition analysis (EDA) method.^19^ The evolution of these EDA terms along the reaction coordinate, once again from the initial stages of the processes up to the respective TSs, is graphically shown in Figure 4a. The differences in Δ*E*_int_ between the uncatalyzed and catalyzed cycloadditions can mainly be assigned to the reduced Pauli repulsion, which is clearly less destabilizing in the latter processes along the entire coordinate. As expected, the situation of the **cat2′**-cycloaddition is an intermediate between the uncatalyzed and the **cat1**-catalyzed reactions. Dispersion interactions (Δ*E*_disp_) are also more stabilizing for the catalyzed reactions, but their contributions are much less significant as compared to the reduction in the Pauli repulsion. At variance, the electrostatic and orbital interactions are similar or even more stabilizing for the uncatalyzed process, and therefore are not at all responsible for the stronger interaction computed for the **cat1**- and **cat2′**-mediated reactions. Therefore, it is confirmed that the Pauli-repulsion lowering concept, which explains the mode of activation of Lewis acids in Diels–Alder reactions,^12^ is also operative in these halogen-bonding-catalyzed cycloadditions. This indicates a similar mode of activation despite the rather different way the catalyst binds to the dienophile: halogen bonding in **cat1** (and **cat2**) vs the dative bond (to the p or d atomic orbital of BF_3_ or TiCl_4_, for instance) in the Lewis acid catalysis.

This Pauli-repulsion lowering is the result of the significant polarization, induced by the catalyst, of the occupied π-molecular orbital on the reactive C=C bond of the dienophile away from the incoming diene. This polarization is reflected in a clear reduction of the orbital overlap (computed at a consistent C···C bond-forming distance of 2.1 Å)^22^ between the key occupied π-molecular orbitals of the diene (highest occupied molecular orbital (HOMO)-1, where all 2p_*z*_ atomic orbitals, located on the reacting C=C double bonds, are in-phase) and the dienophile (where the 2p_*z*_ atomic orbitals located on the reactive C=C double bond are in-phase, Figure 4b).

**Figure 5:**
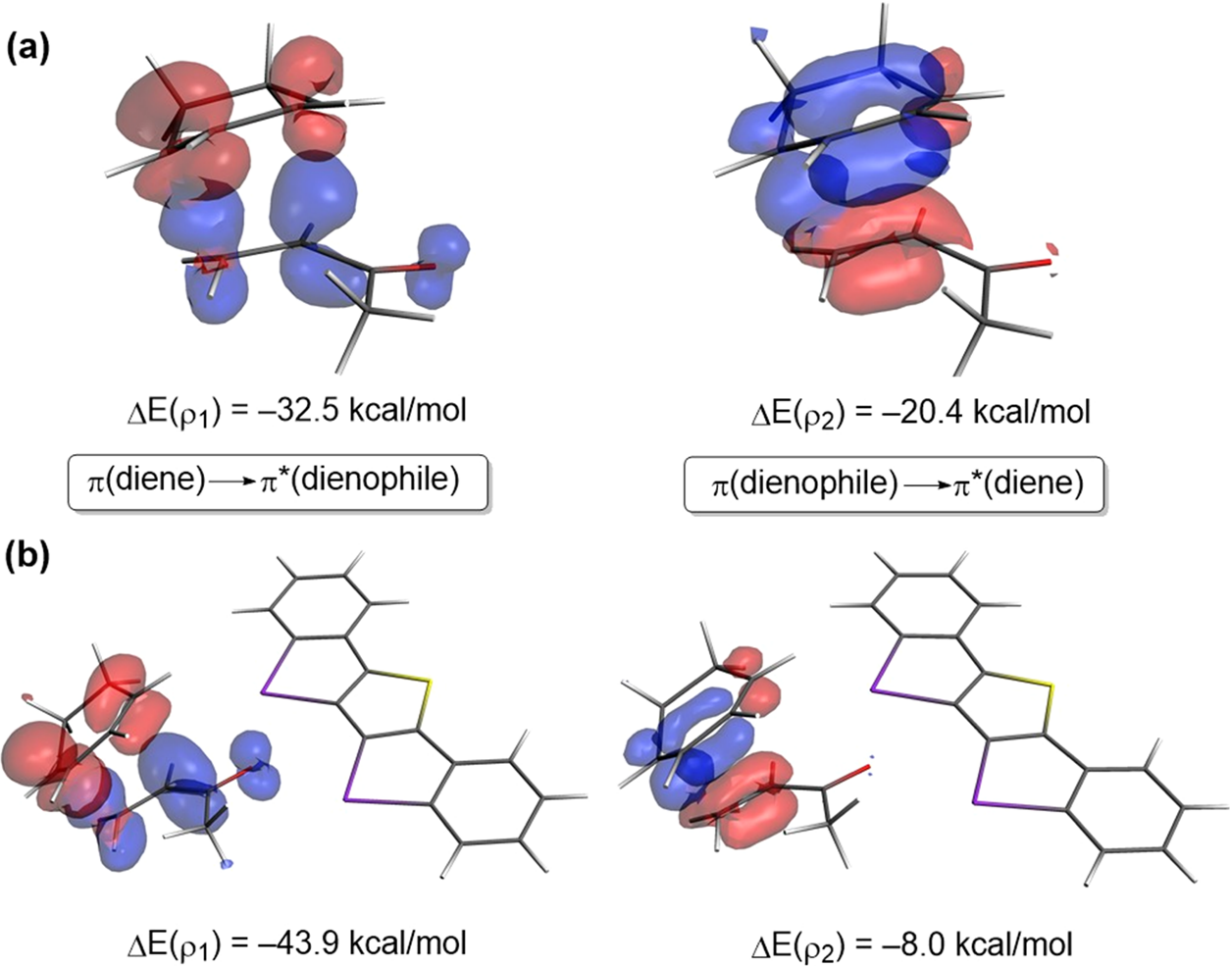
Contour plots of NOCV deformation densities Δρ and associated energies Δ*E*(ρ) (computed at the ZORA-B3LYP-D3/TZ2P//PCM(DCM)-B3LYP-D3/def2-SVP level) for the (a) uncatalyzed and (b) **cat1**-catalyzed DA reactions between cyclohexadiene and methyl vinyl ketone computed at the same consistent C···C bond-forming distance of 2.1 Å. Electron-density charge flow: red → blue.

Finally, the natural orbitals for chemical valence (NOCV)^23^ extension of the EDA method was used to reveal the origin of the counterintuitive finding that the total orbital interactions (Δ*E*_orb_) are not more stabilizing for the catalyzed processes despite benefiting from a more favorable HOMO(diene)–LUMO-π*(dienophile) gap (see above). This approach, for the extreme situations represented by the uncatalyzed and **cat1**-catalyzed cycloadditions, identifies two main orbital interactions that dominate the total orbital interactions, namely, the normal electron demand HOMO(diene) → LUMO-π*(dienophile) and the inverse electron demand LUMO(diene) ← π-HOMO(dienophile) interactions (ρ_1_ and ρ_2_, respectively, Figure 5). Not surprisingly, ρ_1_ > ρ_2_ in both cases, which agrees with the normal electron demand nature of these cycloadditions. Due to the strong electron-withdrawing nature of the iodine(III)-catalyst, which greatly stabilizes the π*-MO of the dienophile, the direct ρ_1_ interaction is much stronger in the **cat1**-catalyzed process than in the parent uncatalyzed reaction (ΔΔ*E*(ρ_1_) = −11.4 kcal/mol, computed at the same consistent C···C bond-forming distance of 2.1 Å).^22^ In addition, the catalyst also weakens the inverse ρ_2_ to a nearly identical extent (ΔΔ*E*(ρ_2_) = +12.4 kcal/mol), which efficiently offsets the stabilization gained in the direct ρ_1_ interaction. For this reason, the total orbital interactions computed for the **cat1**-cycloaddition are not more stabilizing but slightly less stabilizing than those computed for the uncatalyzed process. This result reinforces the generality of the Pauli-repulsion concept^12,14^ rather than the traditional LUMO lowering to rationalize the catalysis in fundamental processes in organic chemistry.

Results above confirmed that the mode of activation of the iodine(III)-based halogen-donor catalyst **cat1** strongly resembles that of strong Lewis acids despite the rather different binding to the dienophile. However, the computed barrier for the analogous cycloaddition reaction between MVK and cyclohexadiene catalyzed by BF_3_ is Δ*G*^≠^ = 16.9 kcal/mol, which, in agreement with the experimental observations,^11^ indicates that the BF_3_-catalyzed reaction is still faster than the **cat1**-catalyzed reaction (Δ*G*^≠^ = 20.1 kcal/mol). At this point, and based on the above-described factors controlling the activity of the halogen-donor catalyst, we hypothesized that an increase in the electrophilic nature of **cat1** should result in a significant increase of its activity (i.e., leading to a lower barrier cycloaddition), which might surpass that of the BF_3_ Lewis acid. To check our hypothesis, we made the aromatic rings bearing the iodine(III) atoms more electron-deficient by replacing their hydrogen atoms with electron-withdrawing groups (F and NO_2_). Table 2 shows the computed barrier and reaction energies for the same cycloaddition reaction (cyclohexadiene + MVK) mediated by these modified **cat1** systems and the EDA–instantaneous interaction energy (Δ*E*_int_) between the catalyst and MVK (**1**) fragments in the reactive **1-cat** complexes. Once again, in all cases, the processes proceed in a concerted manner through the corresponding six-membered transition states (see Figure S2 for a representation of the optimized geometries of these saddle points).

**Table 2:**
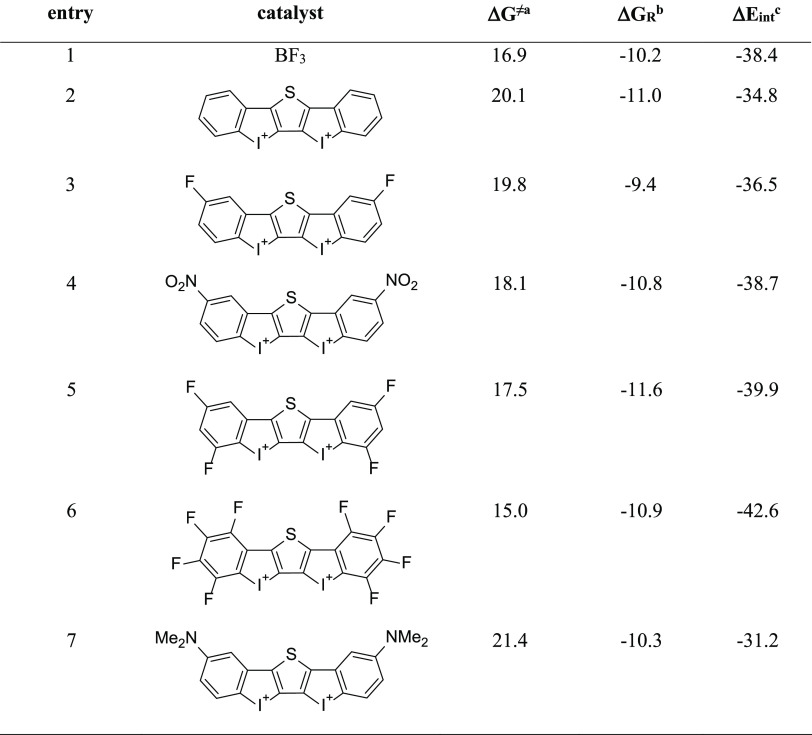
Computed Barrier (Δ*G*^≠^) and Reaction (Δ*G*_R_) Energies (in kcal/mol) for the Catalyzed Cycloaddition Reactions Involving Cyclohexadiene and Methyl Vinyl Ketone^d^

a Δ*G*^≠^ computed as Δ*G*^≠^ = *G*(TS) – *G*(cyclohexadiene) – *G*(MVK-cat complex).

b Δ*G*_R_ computed as Δ*G*_R_ = *G*(cycloadduct) – *G*(cyclohexadiene) – *G*(MVK-cat complex).

c EDA-based interaction energy (Δ*E*_int_, in kcal/mol) between the catalyst and MVK fragments in the corresponding **1-cat** complexes.

d All data have been computed at the PCM(DCM)-B3LYP-D3/def2-TZVPP//PCM(DCM)-B3LYP-D3/def2-SVP level.

From the data in Table 2, it becomes clear that the introduction of two fluorine atoms or two nitro groups at the *para*-position (relative to the iodine atoms) leads to a slight but noticeable decrease of the barrier as compared to **cat1** (ΔΔ*G*^≠^ up to −2 kcal/mol, entry 4). The effect is more pronounced when introducing four fluorine atoms (*para*- and *ortho*-relative positions, ΔΔ*G*^≠^ = −2.6 kcal/mol, entry 5) and even more when all of the aromatic hydrogen atoms were replaced by fluorine atoms (ΔΔ*G*^≠^ = −5.1 kcal/mol, entry 6), which agrees with the reported enhancement of the Lewis acidity in halogen donors by fluorination.^24^ Strikingly, the activity of the latter catalyst (having up to 8 fluorine atoms) surpasses that of the strong Lewis acid BF_3_ (ΔΔ*G*^≠^ = −1.9 kcal/mol), which confirms our hypothesis that the electrophilic nature of the halogen bond donor can be tuned to produce highly active systems. The high electrophilicity of the latter system (**cat1-F8**) is reflected in the strong interaction computed for the corresponding **1**–**cat1-F8** complex (Δ*E*_int_ = −42.6 kcal/mol), which is not only stronger than that in the parent **1-cat1** (Δ*E*_int_ = −34.8 kcal/mol) but also than that of the Lewis acid **1-BF**_**3**_ complex (Δ*E*_int_ = −38.4 kcal/mol). To further support this finding, we calculated the analogous cycloaddition reaction mediated by a system having two electron-donor groups (NMe_2_) in the *para*-position. As expected, the lower electrophilic nature of this organocatalysis (Δ*E*_int_ = −31.2 kcal/mol in the corresponding **1-cat1-NMe**_**2**_ complex) leads to a clear increase of the barrier when compared to the parent system **cat1** (ΔΔ*G*^≠^ = +1.3 kcal/mol, entry 7). Therefore, our calculations indicate that despite these iodine(III)-organocatalysts bind the dienophile through noncovalent halogen bond interactions, their activity can be efficiently modulated to not only approach but also surpass that of covalently bonded Lewis acids.

**Figure 6:**
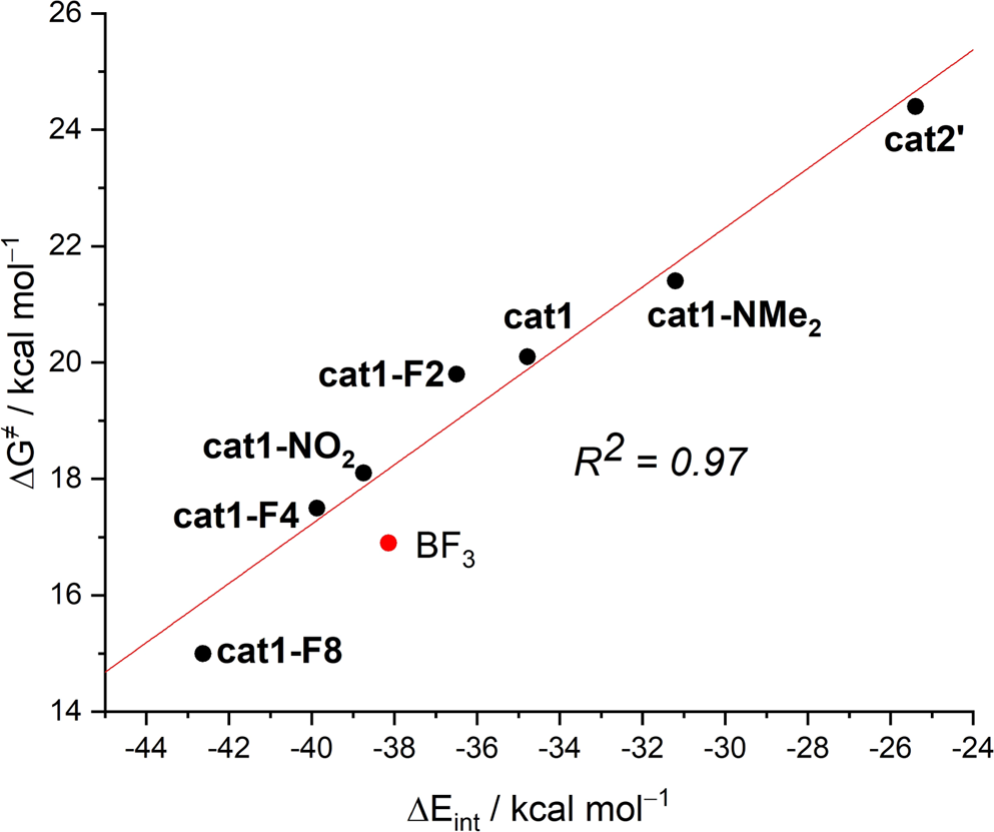
Plot of the activation barriers (Δ*G*^≠^) vs the EDA–instantaneous interaction energies (Δ*E*_int_) in the reactive **1-cat** complexes.

Results above indicate that the trend in reactivity (measured by the computed barrier energies, Δ*G*^≠^) is identical to that of the computed EDA–instantaneous interaction energy (Δ*E*_int_) in the reactive **1-cat** complexes (using MVK (**1**) and the catalyst as fragments). Indeed, a very good linear relationship is found when plotting both parameters (correlation coefficient *R*^2^ = 0.97, see Figure 6), therefore indicating that the strength of the halogen bonding between the catalyst and the Lewis base **1**, measured by the easy-to-compute Δ*E*_int_ values, can be used as a reliable, quantitative measure of the barrier associated with the corresponding Diels–Alder cycloaddition reaction.

**Figure 7:**
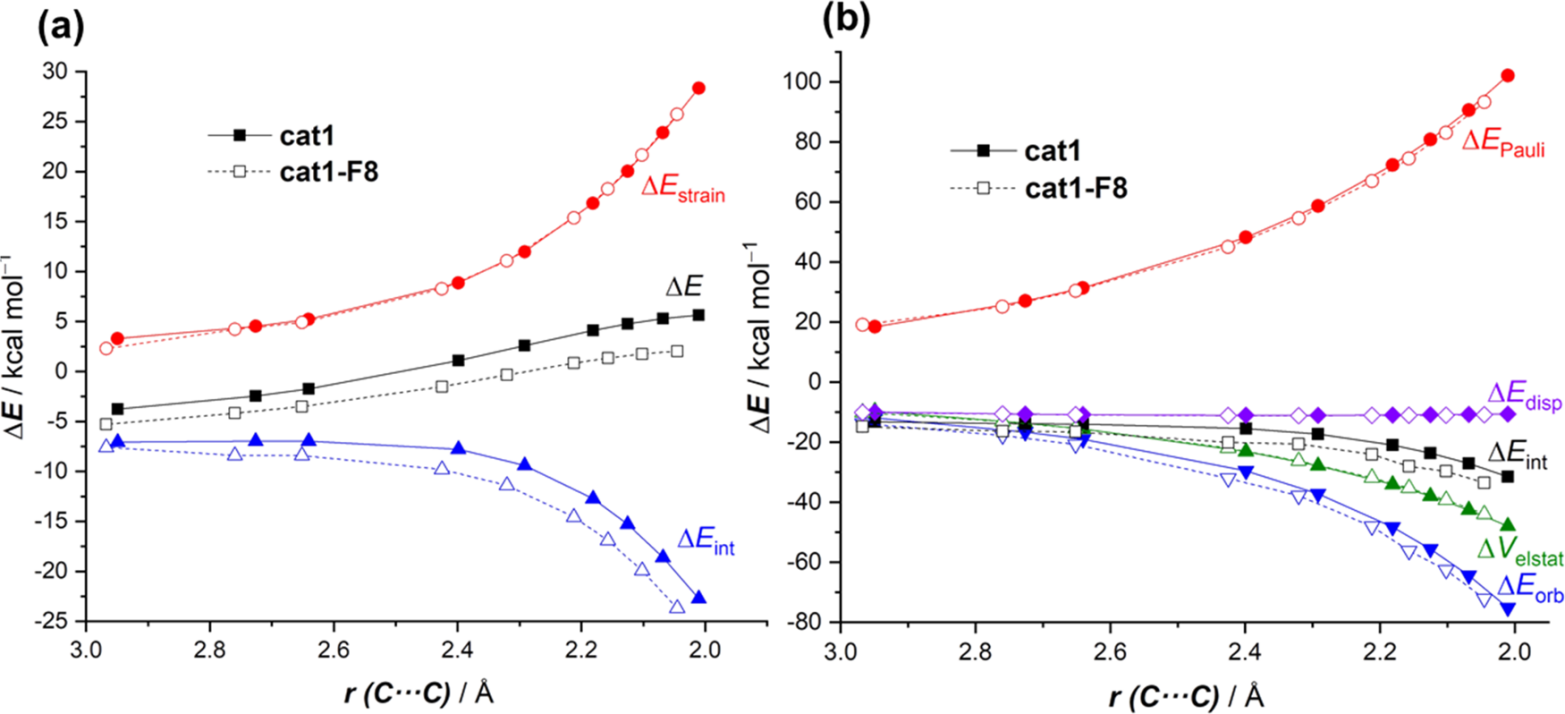
Comparative activation strain analyses (a), computed at the PCM(DCM)-B3LYP-D3/def2-TZVPP//PCM(DCM)-B3LYP-D3/def2-SVP level, and energy decomposition analysis (b), computed at the ZORA-B3LYP-D3/TZ2P//PCM(DCM)-B3LYP-D3/def2-SVP level, of the Diels–Alder reactions between cyclohexadiene and **cat1-** and **cat1-F8**-bonded methyl vinyl ketone complexes projected onto the shorter C···C bond-forming distance.

We applied the ASM approach again to quantitatively understand the reasons behind the remarkable reduction of the barrier of the process mediated by the F8-substituted catalyst (**cat1-F8**) with respect to the parent catalysis **cat1**. The computed ASDs in Figure 7a, once again from the separate reactants to the corresponding transition states, clearly indicate that the lower barrier of the cycloaddition mediated by **cat1-F8** results exclusively from a stronger interaction between the deformed reactants along the entire reaction coordinate. The strain energy, at variance, is identical for both reactions, and therefore, it is not responsible for the activation barrier difference. This identical strain can be ascribed to the almost negligible difference in the asynchronicity in both cycloaddition reactions (**cat1**: Δ*r*_C···C_^TS^ = 0.92 Å; **cat1-F8**: Δ*r*_C···C_^TS^ = 0.95 Å; see Figure S2).

**Figure 8:**
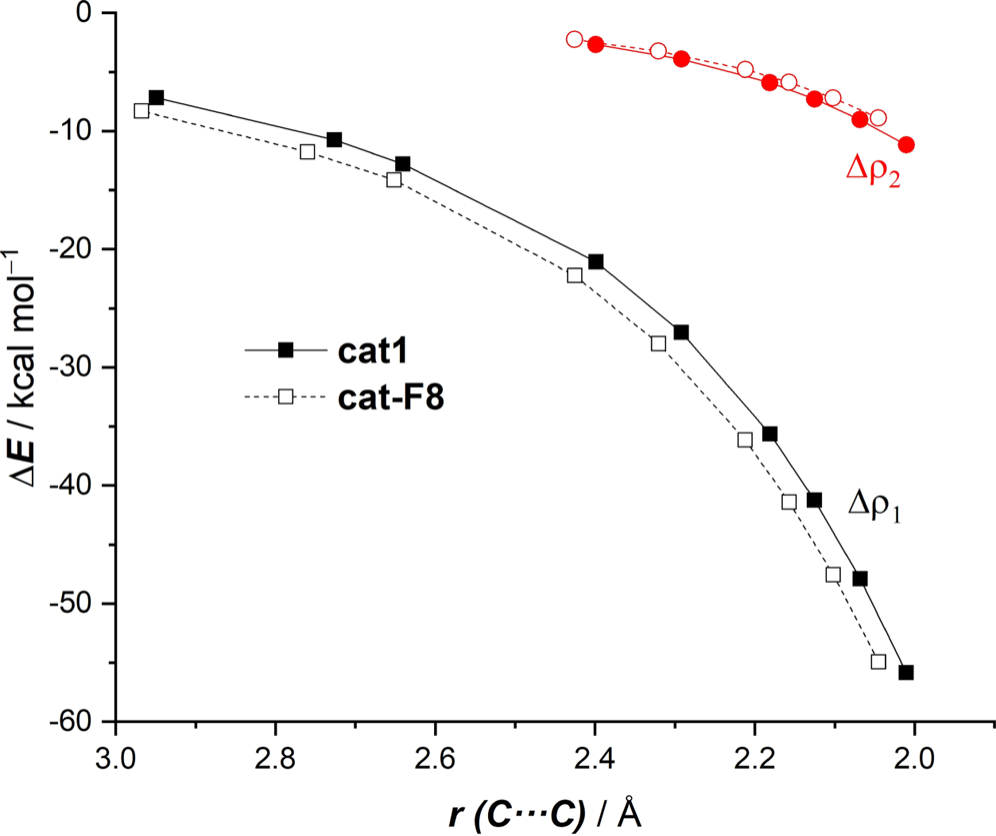
Evolution of the main orbital interactions (ρ_1_ and ρ_2_) involved in the Diels–Alder reactions between cyclohexadiene and **cat1-** and **cat1-F8**-bonded methyl vinyl ketone complexes projected onto the shorter C···C bond-forming distance. All data have been computed at the ZORA-B3LYP-D3/TZ2P//PCM(DCM)-B3LYP-D3/def2-SVP level.

According to Figure 7b, which graphically shows the evolution of the EDA contributors along the reaction coordinate, the stronger (i.e., more stabilizing) interaction energy between the deformed reactants computed for the **cat1-F8**-catalyzed cycloaddition derives solely from the stronger orbital interactions (Δ*E*_orb_) computed for this reaction, as all of the other terms are nearly identical for both processes. For instance, at the same consistent C···C bond-forming distance of 2.1 Å, the difference in the orbital interactions (ΔΔ*E*_orb_ = 3.8 kcal/mol) roughly matches that in the interaction energy (ΔΔ*E*_int_ = 4.4 kcal/mol). The NOCV extension of the EDA method ascribes these enhanced orbital interactions for the **cat1-F8**-mediated process exclusively to the normal electron demand HOMO(diene) → LUMO-π*(dienophile) interaction (ρ_1_), which, as shown in Figure 8, is more stabilizing along the entire reaction coordinate when compared to the analogous process involving **cat1**. Therefore, a further enhancement of the electrophilic nature of the iodine(III)-catalyst results in a stronger HOMO(diene) → LUMO-π*(dienophile) orbital interaction, which ultimately leads to a highly active catalyst whose activity surpasses that of the strong Lewis acid BF_3_.

## Conclusions

From the computational study reported herein, we can conclude that the bidentate iodine(III)-based halogen-donor catalyst **cat1** binds the carbonyl group of the dienophile MVK (**1**) through a double halogen bond interaction, which significantly stabilizes the key π*-molecular orbital (located on the reactive C=C bond) with respect to the parent MVK. This LUMO lowering is, however, not responsible for the acceleration observed in the **cat1**-catalyzed reaction; instead, the organocatalyst induces a remarkable polarization of the occupied π-orbital on the reactive C=C bond of the dienophile away from the incoming diene, which reduces the four-electron Pauli repulsion between the π-systems of the reactants. This Pauli-repulsion lowering effect resembles the way Lewis acids catalyze Diels–Alder cycloaddition reactions despite the fact that the bonding situation of the corresponding reactive dienophile–catalyst complexes is markedly different (i.e., halogen bonding vs the dative bond). Although the activity of the iodine(III)-catalyst is clearly higher than the most active iodine(I)-based halogen donors reported so far (**cat2**), the potency of **cat1** is still lower than that of strong Lewis acids such as BF_3_. Nevertheless, the activity of this species can be further enhanced by increasing the electrophilic nature of the system. Indeed, the replacement of the hydrogen atoms of the aryl groups of the catalyst by electron-withdrawing atoms/groups leads to lower barrier processes. Our calculations suggest that the iodine(III)-catalyst **cat1-F8**, having up to eight fluorine atoms in its structure, constitutes a really promising candidate whose activity is predicted to be even higher than that of the strong Lewis acid BF_3_. Moreover, it is found that the strength of the halogen bonding between the catalyst and the Lewis base **1** can be used as a reliable, quantitative measure of the barrier associated with the corresponding Diels–Alder cycloaddition reaction. The present study not only rationalizes, in a quantitative manner, the so far poorly understood way these halogen-donor systems catalyze Diels–Alder cycloaddition additions (i.e., following the Pauli-repulsion lowering concept) but also provides new insights that can be further applied toward the rational design of highly active catalysts.

## Computational Details

Geometry optimizations of the molecules were performed without symmetry constraints using the Gaussian09 (rev D.01)^25^ suite of programs at the dispersion-corrected B3LYP^26^-D3^27^/def2-SVP^28^ level including solvent effects (solvent = dichloromethane) with the polarization continuum model (PCM) method.^29^ Reactants and adducts were characterized by frequency calculations and have positive definite Hessian matrices. Transition states show only one negative eigenvalue in their diagonalized force constant matrices, and their associated eigenvectors were confirmed to correspond to the motion along the reaction coordinate under consideration using the intrinsic reaction coordinate (IRC) method.^30^ Energy refinements were carried out by means of single-point calculations at the same DFT level using the much larger triple-ζ basis set def2-TZVPP.^28^ This level is denoted as PCM(DCM)-B3LYP-D3/def2-TZVPP//PCM(DCM)-B3LYP-D3/def2-SVP.

### Activation Strain Model of Reactivity and Energy Decomposition Analysis

Within the ASM method,^20^ also known as the distortion/interaction model,^20d^ the potential energy surface Δ*E*(ζ) is decomposed along the reaction coordinate, ζ, into two contributions, namely, the strain Δ*E*_strain_(ζ) associated with the deformation (or distortion) required by the individual reactants during the process and the interaction Δ*E*_int_(ζ) between these increasingly deformed reactants
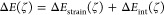


Within the energy decomposition analysis (EDA) method,^19^ the interaction energy can be further decomposed into the following chemically meaningful terms
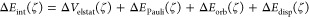


The term Δ*V*_elstat_ corresponds to the classical electrostatic interaction between the unperturbed charge distributions of the deformed reactants and is usually attractive. The Pauli-repulsion Δ*E*_Pauli_ comprises the destabilizing interactions between occupied orbitals and is responsible for any steric repulsion. The orbital interaction Δ*E*_orb_ accounts for bond pair formation, charge transfer (interaction between occupied orbitals on one moiety with unoccupied orbitals on the other, including HOMO–LUMO interactions), and polarization (empty-occupied orbital mixing on one fragment due to the presence of another fragment). Finally, the Δ*E*_disp_ term accounts for the interactions coming from dispersion forces. Note that the concepts of Pauli repulsion and orbital interaction that feature in our canonical EDA have also been successfully applied to reactions that were studied using other decomposition schemes such as DFT-SAPT^31^ or valence bond (VB) theory.^32^ Moreover, the natural orbital for chemical valence (NOCV)^23^ extension of the EDA method has also been used for further partitioning the Δ*E*_orb_ term. The EDA–NOCV approach provides pairwise energy contributions for each pair of interacting orbitals to the total bond energy.

The program package ADF^33^ was used for EDA calculations using the optimized PCM(DCM)-B3LYP-D3/def2-SVP geometries at the same B3LYP-D3 level in conjunction with a triple-ζ-quality basis set using uncontracted Slater-type orbitals (STOs) augmented by two sets of polarization functions with a frozen-core approximation for the core electrons.^34^ Auxiliary sets of s, p, d, f, and g STOs were used to fit the molecular densities and to represent the Coulombic and exchange potentials accurately in each SCF cycle.^35^ Scalar relativistic effects were incorporated by applying the zeroth-order regular approximation (ZORA).^36^ This level of theory is denoted as ZORA-B3LYP-D3/TZ2P//PCM(DCM)-B3LYP-D3/def2-SVP.

## Supporting Information Available

Cartesian coordinates (in Å) and total energies of all of the stationary points discussed in the text (PDF)
