# Supplementary material for: Catalysis by Bidentate Iodine(III)-Based Halogen Donors: Surpassing the Activity of Strong Lewis Acids
Source: J Org Chem. 2021 Mar 25;86(7):5317–26. doi: 10.1021/acs.joc.1c00534 (PMC9132362; doi:10.1021/acs.joc.1c00534)
Supplement: Supplementary file 1 — jo1c00534_si_001.pdf [file jo1c00534_si_001.pdf]

Catalysis by Bidentate Iodine(III)-based Halogen donors: Surpassing the activity of strong Lewis acids

Susana Portela, Jorge J. Cabrera-Trujillo and Israel Fernández*

Departamento de Química Orgánica I and Centro de Innovación en Química Avanzada (ORFEO-CINQA), Facultad de Ciencias Químicas, Universidad Complutense de Madrid, 28040-Madrid (Spain).

E-mail: israel@quim.ucm.es

Contents:

1. Figure S1……S2

2. Figure S2……S3

3. Cartesian coordinates and energies……S4

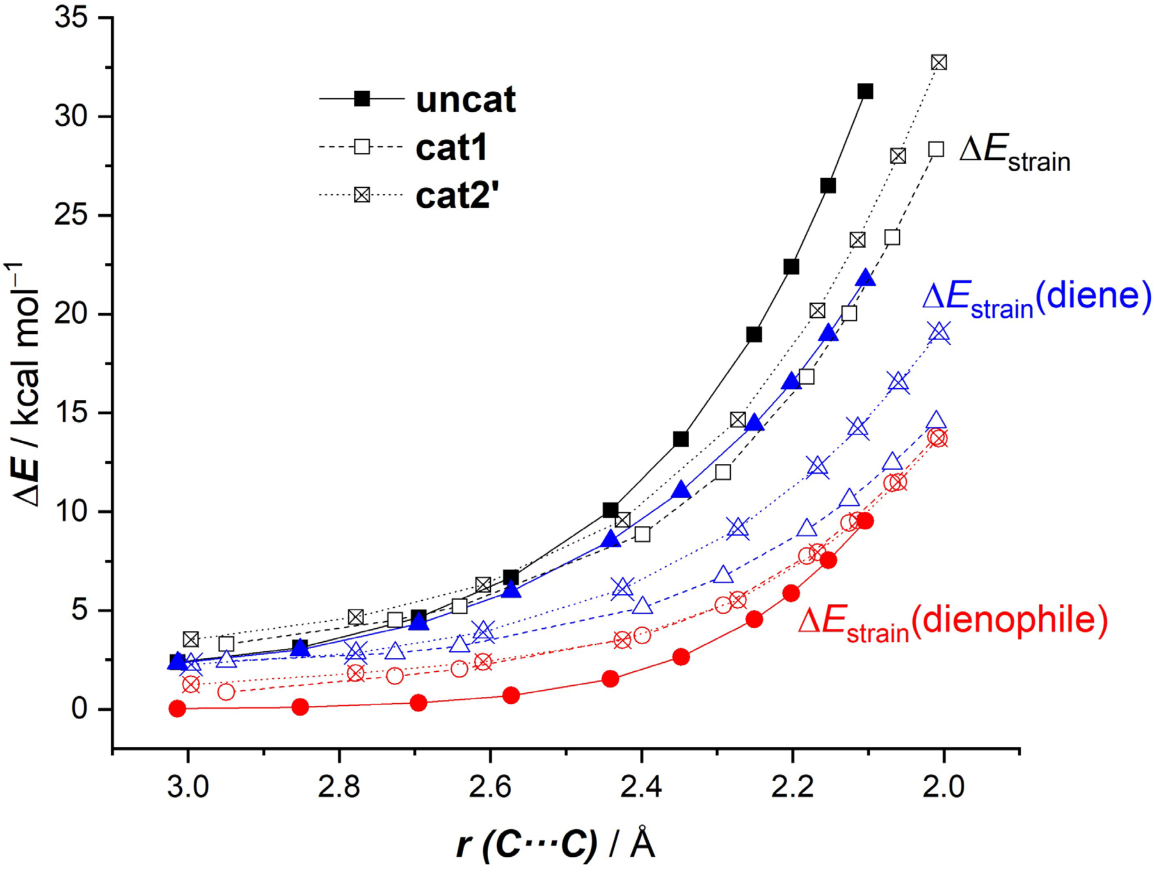

Figure S1. Decomposition of the strain energy associated with the deformation of the diene and dienophile of the Diels-Alder reactions between cyclohexadiene and uncoordinated, **cat1**-, **cat2'**-bonded methyl vinyl ketone complexes projected onto the shorter C···C bond-forming distance.

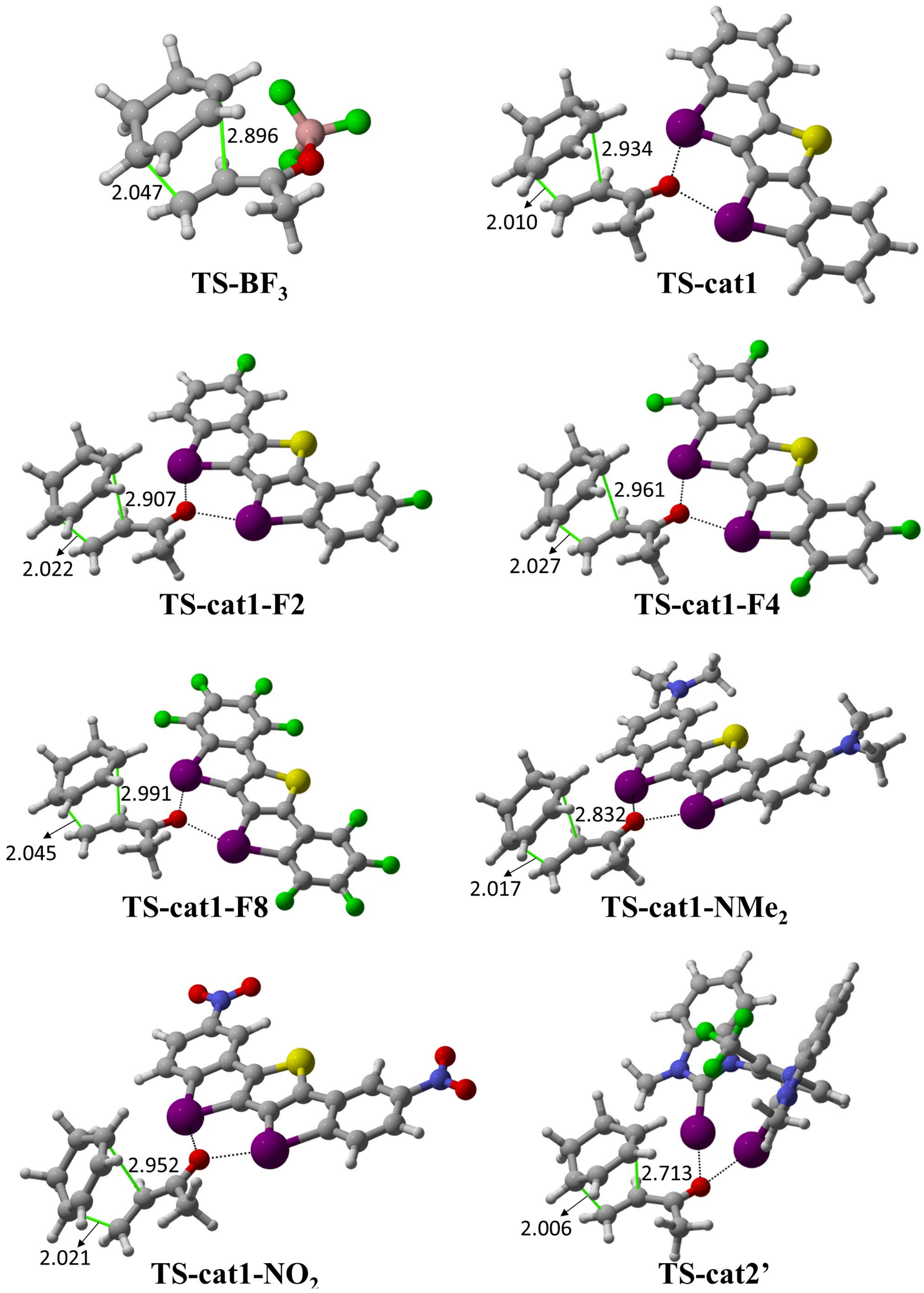

Figure S2. Representation of the optimized transition states (PCM(DCM)-B3LYP-D3/def2-SVP level) for the catalyzed Diels-Alder reactions between cyclohexadiene and methyl vinyl ketone. Bond distances are given is angstroms.

Cartesian coordinates (in Å) and total energies of all the stationary points discussed in the text. All calculations have been performed at the PCM(DCM)-B3LYP-D3/def2-TZVPP//PCM(DCM)-B3LYP-D3/def2-SVP level.

Cyclohexadiene: E= -233.520015028 (i = 0)

C	-0.110675000	1.426692000	-0.065100000
C	1.194877000	0.729988000	0.240734000
C	1.194866000	-0.730007000	-0.240734000
C	-0.110698000	-1.426690000	0.065100000
C	-1.261694000	-0.726979000	0.105563000
C	-1.261682000	0.726999000	-0.105564000
H	1.350732000	-0.758903000	-1.339365000
H	2.045456000	1.276131000	-0.196894000
H	1.350745000	0.758885000	1.339365000
H	-0.113859000	2.514670000	-0.185029000
H	-0.113899000	-2.514667000	0.185032000
H	-2.217299000	-1.233046000	0.272951000
H	-2.217279000	1.233082000	-0.272951000
H	2.045436000	-1.276164000	0.196892000

1, Methyl vinyl ketone (MVK): E= -231.345421666 (i = 0)

C	-0.871976000	-0.637105000	0.000181000
H	-0.985906000	-1.726410000	0.000485000
C	-1.943770000	0.167601000	-0.000129000
H	-2.958108000	-0.241204000	-0.000070000
H	-1.855743000	1.257719000	-0.000426000
C	0.548140000	-0.186985000	0.000012000
C	0.850995000	1.297983000	0.000080000
H	0.414230000	1.784682000	-0.886623000
H	0.414665000	1.784365000	0.887171000
H	1.938808000	1.443040000	-0.000226000
O	1.441465000	-1.018895000	-0.000147000

Complex 1-BF$_3$: E= -556.088498994 (i = 0)

C	-1.404558000	-0.871751000	0.000015000
H	-0.595040000	-1.601319000	0.000144000
C	-2.688733000	-1.271347000	-0.000035000
H	-2.936182000	-2.336027000	0.000173000
H	-3.529734000	-0.573385000	-0.000061000
C	-1.012530000	0.537130000	-0.000177000
C	-2.022448000	1.639355000	0.000034000
H	-2.670520000	1.560552000	-0.886523000
H	-2.670035000	1.560407000	0.886947000
H	-1.511319000	2.608901000	-0.000024000
O	0.194439000	0.878868000	-0.000429000
B	1.481007000	-0.085137000	0.000020000
F	1.384698000	-0.838081000	1.146571000
F	1.384869000	-0.839075000	-1.145893000
F	2.532867000	0.785521000	-0.000273000

Complex 1-cat1: E= -1839.44481678 (i = 0)

C	-1.416775000	4.404069000	0.449113000
H	-2.200188000	3.732351000	0.809938000
C	-1.614925000	5.732749000	0.483492000
H	-2.553503000	6.143390000	0.864680000
H	-0.868301000	6.453561000	0.142059000
C	-0.187984000	3.748164000	-0.017515000
C	1.026294000	4.547144000	-0.396758000
H	0.777614000	5.532103000	-0.809668000

H	1.632962000	4.709134000	0.511218000
H	1.628220000	3.993091000	-1.128784000
O	-0.141587000	2.504222000	-0.055607000
C	-2.521308000	-2.252029000	0.007988000
C	-3.364887000	-1.122088000	-0.037403000
C	-4.750073000	-1.184698000	-0.048751000
C	-5.330195000	-2.459764000	-0.015102000
C	-4.529084000	-3.609045000	0.028883000
C	-3.137774000	-3.515332000	0.040723000
C	-1.098735000	-1.969724000	0.015469000
H	-5.370706000	-0.287630000	-0.082300000
H	-6.418313000	-2.549005000	-0.023380000
H	-4.999041000	-4.593916000	0.054969000
H	-2.524069000	-4.418197000	0.075757000
C	-0.640068000	-0.663342000	-0.018407000
C	3.530207000	-0.661369000	0.023151000
C	2.842155000	-1.892372000	0.052360000
C	3.616500000	-3.064325000	0.107409000
C	5.007781000	-2.976044000	0.134351000
C	5.652067000	-1.731529000	0.111418000
C	4.911382000	-0.543265000	0.055776000
C	0.768253000	-0.568040000	-0.013339000
C	1.395704000	-1.801422000	0.030925000
H	3.122731000	-4.038102000	0.131507000
H	5.601824000	-3.890791000	0.176989000
H	6.742203000	-1.677401000	0.138005000
H	5.410422000	0.426958000	0.043035000
S	0.230207000	-3.094318000	0.061222000
I	2.168106000	0.991422000	-0.073448000
I	-2.233836000	0.696000000	-0.066201000

Complex 1-cat1-F2: E= -2038.00076081 (i = 0)

C	-1.440917000	4.698714000	-0.121121000
H	-2.318495000	4.059116000	-0.242419000
C	-1.603737000	6.032586000	-0.084490000
H	-2.601490000	6.470000000	-0.173232000
H	-0.772648000	6.730915000	0.036173000
C	-0.155411000	4.000463000	-0.012250000
C	1.149101000	4.742049000	0.068839000
H	1.044049000	5.823277000	0.201136000
H	1.740104000	4.337229000	0.903845000
H	1.707114000	4.561177000	-0.865220000
O	-0.137490000	2.753553000	-0.005860000
C	-2.559027000	-1.969415000	-0.009731000
C	-3.387522000	-0.826911000	0.016317000
C	-4.774966000	-0.882454000	0.037930000
C	-5.373712000	-2.145209000	0.035508000
C	-4.567876000	-3.287993000	0.010448000
C	-3.177720000	-3.229022000	-0.012878000
C	-1.133244000	-1.703285000	-0.025552000
H	-6.459151000	-2.253767000	0.053149000
H	-2.599144000	-4.154015000	-0.032073000
C	-0.662475000	-0.400383000	-0.016623000
C	3.502567000	-0.442082000	0.013812000
C	2.804366000	-1.667959000	-0.022689000
C	3.555438000	-2.852929000	-0.038204000
C	4.943653000	-2.762104000	-0.013223000
C	5.620135000	-1.539083000	0.029864000
C	4.887765000	-0.349037000	0.044231000
C	0.746148000	-0.320169000	-0.018984000
C	1.358648000	-1.562020000	-0.032933000

H	3.078768000	-3.833708000	-0.069409000
H	6.710774000	-1.529201000	0.051689000
S	0.181617000	-2.842741000	-0.043216000
I	2.160326000	1.226534000	0.017297000
I	-2.240626000	0.976232000	0.017211000
F	5.656459000	-3.886660000	-0.029162000
F	-5.154182000	-4.483722000	0.008900000
H	-5.391543000	0.017745000	0.057008000
H	5.403839000	0.610966000	0.081471000

Complex 1-cat1-F4: E= -2236.54952859 (i = 0)

C	-1.258998000	4.640209000	0.451029000
H	-2.089484000	4.012997000	0.784803000
C	-1.400582000	5.977476000	0.452029000
H	-2.338577000	6.433990000	0.778341000
H	-0.609617000	6.659146000	0.131413000
C	-0.046976000	3.924730000	0.044809000
C	1.233438000	4.642222000	-0.267207000
H	1.106841000	5.712771000	-0.458634000
H	1.906933000	4.532381000	0.600225000
H	1.713884000	4.174153000	-1.138301000
O	-0.065405000	2.676057000	-0.008946000
C	-2.610545000	-1.938253000	0.022621000
C	-3.382819000	-0.760107000	-0.042268000
C	-4.771587000	-0.791604000	-0.075293000
C	-5.433052000	-2.015167000	-0.050003000
C	-4.663970000	-3.181432000	0.011305000
C	-3.270711000	-3.174564000	0.049524000
C	-1.177910000	-1.709063000	0.047102000
H	-6.522337000	-2.058595000	-0.077664000
H	-2.727175000	-4.119067000	0.098632000
C	-0.674914000	-0.418331000	0.011302000
C	3.457365000	-0.553347000	-0.014718000
C	2.757729000	-1.775246000	0.056433000
C	3.492971000	-2.967440000	0.109768000
C	4.884379000	-2.888944000	0.087555000
C	5.581198000	-1.678746000	0.014933000
C	4.845173000	-0.499487000	-0.035553000
C	0.734806000	-0.375409000	0.014740000
C	1.313726000	-1.633532000	0.061363000
H	3.009056000	-3.942799000	0.170123000
H	6.671298000	-1.655803000	-0.001125000
S	0.104127000	-2.881198000	0.100330000
I	2.182436000	1.142431000	-0.078704000
I	-2.211234000	1.009374000	-0.063698000
F	5.585796000	-4.016142000	0.137951000
F	-5.294961000	-4.350401000	0.034257000
F	-5.454681000	0.347630000	-0.131464000
F	5.453886000	0.680254000	-0.104191000

Complex 1-cat1-F8: E= -2633.61344329 (i = 0)

C	1.232026000	4.939663000	-0.304056000
H	2.012921000	4.348317000	-0.789299000
C	1.408482000	6.263816000	-0.145656000
H	2.327322000	6.743750000	-0.492149000
H	0.666495000	6.909748000	0.328848000
C	0.041195000	4.197229000	0.108873000
C	-1.196662000	4.887178000	0.599418000
H	-1.021813000	5.916952000	0.928512000
H	-1.925528000	4.921672000	-0.229328000
H	-1.637945000	4.313568000	1.426865000

O	0.044222000	2.948118000	0.012381000
C	2.622264000	-1.606482000	0.036008000
C	3.401240000	-0.431889000	-0.004370000
C	4.786151000	-0.450405000	-0.029401000
C	5.437073000	-1.692072000	-0.012947000
C	4.690623000	-2.877869000	0.027299000
C	3.294532000	-2.833798000	0.051747000
C	1.190654000	-1.403995000	0.048352000
C	0.682959000	-0.114285000	0.026312000
C	-3.454044000	-0.263550000	-0.026216000
C	-2.735082000	-1.475483000	0.013852000
C	-3.467976000	-2.667548000	0.012984000
C	-4.864326000	-2.641470000	-0.028473000
C	-5.550295000	-1.419422000	-0.068666000
C	-4.837221000	-0.212464000	-0.067567000
C	-0.725576000	-0.079695000	0.022957000
C	-1.295382000	-1.343278000	0.037963000
S	-0.082540000	-2.589691000	0.068803000
I	-2.177177000	1.435432000	-0.008838000
I	2.208986000	1.327269000	-0.039176000
F	-5.546553000	-3.771714000	-0.031194000
F	5.316299000	-4.040146000	0.040514000
F	5.492369000	0.669626000	-0.070467000
F	-5.483588000	0.943446000	-0.106872000
F	-2.832650000	-3.833041000	0.047074000
F	-6.869727000	-1.407356000	-0.108578000
F	6.755750000	-1.747712000	-0.036855000
F	2.600953000	-3.965560000	0.086482000

Complex 1-cat1-NMe$_2$: E= -2107.51386766 (i = 0)

C	1.359945000	5.297929000	-0.082229000
H	2.237295000	4.685867000	-0.306774000
C	1.501679000	6.627260000	0.053613000
H	2.484439000	7.093067000	-0.056123000
H	0.666764000	7.294105000	0.280099000
C	0.096223000	4.560773000	0.048628000
C	-1.220407000	5.261495000	0.240365000
H	-1.138870000	6.342260000	0.392601000
H	-1.837485000	5.084419000	-0.656055000
H	-1.739902000	4.816334000	1.102608000
O	0.109450000	3.318433000	-0.017966000
C	2.619807000	-1.394967000	0.009973000
C	3.425852000	-0.238201000	-0.011947000
C	4.813667000	-0.301455000	-0.020322000
C	5.419890000	-1.554181000	-0.007046000
C	4.656673000	-2.761996000	0.014186000
C	3.239796000	-2.647116000	0.022905000
C	1.189712000	-1.145448000	0.014593000
H	6.507454000	-1.588624000	-0.013716000
H	2.610010000	-3.534393000	0.039366000
C	0.694739000	0.147617000	-0.000839000
C	-3.470673000	0.028204000	-0.022291000
C	-2.754545000	-1.185948000	0.002188000
C	-3.464438000	-2.389271000	0.012281000
C	-4.885840000	-2.399808000	-0.001587000
C	-5.556712000	-1.138757000	-0.027962000
C	-4.859445000	0.065460000	-0.038040000
C	-0.717339000	0.203792000	-0.002911000
C	-1.310341000	-1.046778000	0.010367000
H	-2.898737000	-3.318970000	0.029796000
H	-6.643739000	-1.092671000	-0.040216000

S	-0.108566000	-2.306045000	0.029621000
I	-2.165623000	1.722219000	-0.027139000
I	2.256802000	1.546721000	-0.035784000
H	5.431778000	0.598135000	-0.037339000
H	-5.410746000	1.006443000	-0.058614000
N	-5.584240000	-3.572519000	0.009604000
C	-7.039732000	-3.561699000	-0.016156000
H	-7.461547000	-3.044770000	0.863313000
H	-7.429528000	-3.068944000	-0.923800000
H	-7.409316000	-4.593316000	-0.008944000
N	5.265357000	-3.983910000	0.025508000
C	4.463791000	-5.197861000	0.044844000
H	3.824075000	-5.251937000	0.943348000
H	3.812770000	-5.273116000	-0.843996000
H	5.126125000	-6.070882000	0.050985000
C	-4.874730000	-4.841983000	0.043579000
H	-4.241673000	-4.931998000	0.943704000
H	-5.599338000	-5.663886000	0.059366000
H	-4.230536000	-4.975173000	-0.843375000
C	6.717736000	-4.081507000	0.014851000
H	7.153073000	-3.620940000	-0.889094000
H	7.167698000	-3.596056000	0.898297000
H	7.009234000	-5.137823000	0.027245000

Complex 1-cat1-NO$_2$: E= -2248.61487601 (i = 0)

C	-1.308435000	5.122879000	0.470437000
H	-2.133091000	4.486492000	0.801392000
C	-1.459549000	6.458928000	0.483546000
H	-2.399497000	6.905923000	0.817348000
H	-0.674693000	7.149021000	0.165962000
C	-0.092929000	4.418240000	0.053722000
C	1.180306000	5.147255000	-0.263149000
H	1.046640000	6.219726000	-0.438056000
H	1.865367000	5.027672000	0.593717000
H	1.651230000	4.692849000	-1.146540000
O	-0.102202000	3.170578000	-0.006648000
C	-2.613370000	-1.463658000	0.002254000
C	-3.423795000	-0.308474000	-0.052876000
C	-4.811259000	-0.335499000	-0.081375000
C	-5.434179000	-1.586787000	-0.058609000
C	-4.645731000	-2.738915000	-0.006636000
C	-3.255627000	-2.710066000	0.025286000
C	-1.183149000	-1.224602000	0.023215000
H	-5.411237000	0.574730000	-0.120171000
H	-6.519888000	-1.672162000	-0.080733000
H	-2.694111000	-3.643659000	0.066537000
C	-0.690260000	0.069572000	-0.006146000
C	3.474721000	-0.054797000	-0.043205000
C	2.749284000	-1.264005000	0.025035000
C	3.478401000	-2.460523000	0.073549000
C	4.866959000	-2.392137000	0.047428000
C	5.571397000	-1.188021000	-0.024422000
C	4.860640000	0.014793000	-0.070163000
C	0.719581000	0.122947000	-0.004687000
C	1.305953000	-1.130885000	0.033706000
H	2.983200000	-3.429416000	0.133505000
H	6.660522000	-1.196069000	-0.043039000
H	5.393678000	0.963978000	-0.126355000
S	0.106938000	-2.388758000	0.066117000
I	2.163887000	1.640645000	-0.092310000
I	-2.242255000	1.475494000	-0.068444000

N	5.633284000	-3.663189000	0.099533000
O	6.848466000	-3.586047000	0.079411000
O	4.996081000	-4.700314000	0.158732000
N	-5.320105000	-4.062083000	0.016888000
O	-4.610960000	-5.052614000	0.049917000
O	-6.537837000	-4.070965000	0.001356000

Complex 1-cat2': E= -2232.45389759 (i = 0)

C	-1.913429000	5.450375000	0.020720000
H	-2.709776000	4.699529000	0.015754000
C	-2.209772000	6.758587000	-0.008849000
H	-3.248576000	7.097520000	-0.038393000
H	-1.440616000	7.535547000	-0.005013000
C	-0.543577000	4.900805000	0.062934000
C	0.644640000	5.825251000	0.075057000
H	0.634549000	6.473004000	-0.815283000
H	0.606301000	6.482850000	0.957537000
H	1.576236000	5.246392000	0.093817000
O	-0.387785000	3.676170000	0.089019000
H	-1.990338000	-1.416966000	3.063151000
C	-1.037041000	-1.381813000	2.534093000
C	1.348283000	-1.206127000	1.125572000
C	-1.037145000	-1.484863000	1.139657000
C	0.159226000	-1.211426000	3.226380000
C	1.353836000	-1.097720000	2.516245000
C	0.162471000	-1.451461000	0.406141000
H	0.157298000	-1.132838000	4.314397000
H	2.297980000	-0.909966000	3.029325000
N	2.591432000	-1.008749000	0.434391000
N	-2.313746000	-1.551014000	0.489097000
C	-2.977553000	-0.486017000	-0.031218000
N	-4.135719000	-0.905899000	-0.560926000
C	-3.073583000	-2.704358000	0.281561000
C	-4.234877000	-2.290023000	-0.388831000
C	3.044911000	0.187378000	-0.029730000
N	4.268935000	0.021121000	-0.550063000
C	3.576757000	-1.978941000	0.221259000
C	4.642221000	-1.319524000	-0.409738000
C	-5.163484000	-0.103827000	-1.219514000
H	-6.109025000	-0.209017000	-0.670447000
H	-4.860926000	0.948359000	-1.230955000
H	-5.291913000	-0.460454000	-2.250362000
C	5.126105000	1.033768000	-1.161374000
H	5.440545000	0.681351000	-2.152708000
H	4.571286000	1.971570000	-1.268136000
H	6.009217000	1.195869000	-0.528186000
C	-5.228128000	-3.198778000	-0.765630000
C	-2.838285000	-4.042177000	0.603841000
C	3.617948000	-3.342522000	0.517487000
C	5.809392000	-1.995806000	-0.777356000
H	-6.131165000	-2.880511000	-1.286899000
H	-1.929348000	-4.353925000	1.119400000
H	6.638858000	-1.484570000	-1.266224000
H	2.782381000	-3.846540000	1.003641000
C	-4.999772000	-4.535589000	-0.442517000
C	-3.827640000	-4.949519000	0.228240000
C	4.781201000	-4.017864000	0.152509000
C	5.856055000	-3.357381000	-0.482764000
H	-5.746210000	-5.283389000	-0.717149000
H	-3.692807000	-6.008398000	0.457437000
H	6.747106000	-3.928761000	-0.750020000

H	4.863612000	-5.086096000	0.362261000
C	0.250571000	-1.696597000	-1.097234000
I	-2.184881000	1.451754000	0.003227000
I	1.907187000	1.938130000	0.073639000
F	1.104654000	-2.703817000	-1.342889000
F	0.709545000	-0.616612000	-1.750877000
F	-0.921633000	-2.025677000	-1.649805000

TS-1: E= -464.838812507 (i = -457 cm^{-1})

C	-0.530452000	1.070230000	0.938940000
C	-1.663803000	1.481853000	0.031638000
C	-2.382527000	0.272366000	-0.630814000
C	-1.732439000	-1.058945000	-0.290559000
C	-1.206780000	-1.233332000	0.995416000
C	-0.565977000	-0.140365000	1.609474000
H	-3.423807000	0.223336000	-0.271210000
H	-2.383669000	2.036750000	0.659560000
H	-1.312676000	2.206689000	-0.717445000
H	0.137119000	1.862816000	1.287791000
H	-2.159242000	-1.931731000	-0.795159000
H	-1.074054000	-2.239761000	1.403250000
H	0.073424000	-0.303743000	2.481379000
H	-2.446367000	0.398596000	-1.722459000
C	0.754712000	0.264359000	-1.013193000
H	0.567840000	1.226110000	-1.495535000
C	0.031974000	-0.865347000	-1.419830000
H	-0.546378000	-0.795053000	-2.344144000
H	0.438711000	-1.858952000	-1.224346000
C	2.004082000	0.231069000	-0.240492000
C	2.436068000	-1.092180000	0.370884000
H	1.604845000	-1.603548000	0.878793000
H	2.795104000	-1.767597000	-0.423916000
H	3.254930000	-0.911953000	1.079870000
O	2.700634000	1.234975000	-0.113152000

TS-BF_3: E= -789.604045439 (i = -353 cm^{-1})

C	-1.067085000	-0.823379000	-1.342786000
C	-1.354660000	-1.887803000	-0.330476000
C	-2.476649000	-1.525602000	0.676878000
C	-2.909617000	-0.070146000	0.616850000
C	-2.876156000	0.591612000	-0.614659000
C	-1.907723000	0.232911000	-1.584640000
H	-3.377321000	-2.123856000	0.460749000
H	-1.640112000	-2.788982000	-0.904388000
H	-0.410607000	-2.160088000	0.160545000
H	-0.193146000	-0.983936000	-1.978757000
H	-3.702487000	0.213994000	1.315441000
H	-3.462952000	1.504147000	-0.753842000
H	-1.736550000	0.886112000	-2.443343000
H	-2.179920000	-1.789485000	1.703081000
C	-0.139416000	0.532363000	1.041896000
H	0.309976000	-0.384814000	1.417194000
C	-1.390070000	0.929629000	1.555339000
H	-1.684760000	0.487757000	2.510715000
H	-1.726290000	1.960248000	1.431379000
C	0.601273000	1.259722000	0.090035000
C	0.134290000	2.574341000	-0.465384000
H	-0.957786000	2.649612000	-0.531790000
H	0.488616000	3.385210000	0.194057000
H	0.575640000	2.728168000	-1.458788000
O	1.776181000	0.908292000	-0.317492000

B	2.510568000	-0.377678000	0.038187000
F	2.661641000	-0.454505000	1.417894000
F	1.749223000	-1.451437000	-0.435633000
F	3.728059000	-0.298937000	-0.607992000

TS-cat1: E= -2072.95584124 (i = -384 cm^{-1})

C	-4.048166000	-0.785448000	1.653860000
C	-4.885235000	0.259720000	0.978902000
C	-6.010218000	-0.307851000	0.074703000
C	-5.927866000	-1.809620000	-0.143508000
C	-5.418013000	-2.611821000	0.883842000
C	-4.427653000	-2.097726000	1.757555000
H	-6.988832000	-0.118598000	0.544314000
H	-5.324940000	0.871073000	1.788318000
H	-4.236890000	0.962787000	0.434664000
H	-3.170223000	-0.437582000	2.207006000
H	-6.731583000	-2.235293000	-0.751672000
H	-5.636993000	-3.683332000	0.892322000
H	-3.877875000	-2.779393000	2.409985000
H	-6.035664000	0.213976000	-0.893298000
C	-3.295904000	-1.307955000	-1.133142000
H	-3.279939000	-0.252677000	-1.410652000
C	-4.447277000	-2.049427000	-1.482154000
H	-5.068926000	-1.616651000	-2.270766000
H	-4.388559000	-3.137696000	-1.536817000
C	-2.127599000	-1.822404000	-0.528134000
C	-2.014149000	-3.274454000	-0.134687000
H	-2.983586000	-3.773602000	-0.026750000
H	-1.443587000	-3.818722000	-0.907058000
H	-1.474005000	-3.356179000	0.819180000
O	-1.086519000	-1.091420000	-0.346966000
C	1.093247000	3.555790000	0.071319000
C	-0.307655000	3.536169000	-0.101425000
C	-1.088591000	4.682811000	-0.135083000
C	-0.436497000	5.914202000	0.012681000
C	0.953078000	5.971051000	0.186845000
C	1.718911000	4.806110000	0.216799000
C	1.733391000	2.254442000	0.082832000
H	-2.170256000	4.641435000	-0.271911000
H	-1.024273000	6.834133000	-0.009096000
H	1.444454000	6.939159000	0.301064000
H	2.801372000	4.858463000	0.354071000
C	0.980399000	1.102660000	-0.058363000
C	3.472464000	-2.223612000	0.037070000
C	4.045544000	-0.940780000	0.175446000
C	5.440452000	-0.860104000	0.329745000
C	6.205129000	-2.026140000	0.336141000
C	5.602240000	-3.282991000	0.190047000
C	4.213875000	-3.396759000	0.038256000
C	1.753934000	-0.075067000	-0.026590000
C	3.106968000	0.164208000	0.133840000
H	5.918301000	0.115607000	0.443250000
H	7.288044000	-1.956657000	0.455321000
H	6.213584000	-4.187676000	0.193744000
H	3.746562000	-4.376553000	-0.074892000
S	3.425163000	1.873436000	0.252750000
I	1.334373000	-2.115717000	-0.159000000
I	-1.051269000	1.527044000	-0.302498000

TS-cat1-F2: E= -2271.51275295 (i = -372 cm^{-1})

C	-4.060284000	-1.207641000	1.792546000
C	-4.979123000	-0.097759000	1.377567000
C	-6.197992000	-0.559437000	0.536128000
C	-6.129986000	-2.013572000	0.101221000
C	-5.503064000	-2.938960000	0.943320000
C	-4.421562000	-2.529362000	1.761859000
H	-7.117194000	-0.459946000	1.135662000
H	-5.327046000	0.376668000	2.313519000
H	-4.405072000	0.693658000	0.872665000
H	-3.119392000	-0.919074000	2.271562000
H	-6.989989000	-2.368556000	-0.474431000
H	-5.714858000	-4.005643000	0.827757000
H	-3.796267000	-3.282743000	2.245536000
H	-6.339848000	0.091178000	-0.339334000
C	-3.618058000	-1.314187000	-1.078705000
H	-3.643739000	-0.229105000	-1.190498000
C	-4.793763000	-2.020624000	-1.415773000
H	-5.501169000	-1.490243000	-2.059192000
H	-4.735562000	-3.088211000	-1.633291000
C	-2.392250000	-1.887911000	-0.670027000
C	-2.238682000	-3.379938000	-0.511264000
H	-3.193686000	-3.910578000	-0.436102000
H	-1.697027000	-3.783650000	-1.383678000
H	-1.655435000	-3.599063000	0.394137000
O	-1.340272000	-1.174224000	-0.481143000
C	0.869958000	3.451838000	0.047834000
C	-0.525397000	3.437228000	-0.168783000
C	-1.294794000	4.593131000	-0.219853000
C	-0.649338000	5.820835000	-0.047993000
C	0.732021000	5.846147000	0.167468000
C	1.505777000	4.691044000	0.219254000
C	1.503077000	2.146980000	0.068356000
H	-1.204230000	6.759594000	-0.079835000
H	2.580888000	4.766602000	0.390764000
C	0.745994000	1.002195000	-0.108574000
C	3.211355000	-2.336088000	-0.007532000
C	3.790842000	-1.060932000	0.174672000
C	5.178136000	-0.983016000	0.370733000
C	5.916237000	-2.162192000	0.371637000
C	5.332489000	-3.418863000	0.184475000
C	3.951243000	-3.512175000	-0.008973000
C	1.511964000	-0.179155000	-0.073808000
C	2.861339000	0.051524000	0.128151000
H	5.685388000	-0.028491000	0.519964000
H	5.962589000	-4.309478000	0.191773000
S	3.186119000	1.755531000	0.281124000
I	1.084790000	-2.212875000	-0.265216000
I	-1.275262000	1.438562000	-0.400057000
F	7.234179000	-2.089315000	0.557216000
F	1.332441000	7.025059000	0.330235000
H	-2.371923000	4.562956000	-0.390185000
H	3.486483000	-4.488357000	-0.156834000

TS-cat1-F4: E= -2470.06394323 (i = -362 cm^{-1})

C	-3.844558000	-2.002541000	1.707850000
C	-4.956830000	-1.256816000	1.033788000
C	-5.887961000	-2.139831000	0.163774000
C	-5.371846000	-3.550825000	-0.056961000
C	-4.614191000	-4.157543000	0.949133000
C	-3.806282000	-3.367307000	1.807185000

H	-6.864935000	-2.245654000	0.662585000
H	-5.539151000	-0.775682000	1.840806000
H	-4.546073000	-0.410312000	0.462555000
H	-3.105287000	-1.405970000	2.252006000
H	-6.023160000	-4.200251000	-0.649317000
H	-4.502112000	-5.245256000	0.959629000
H	-3.066330000	-3.851651000	2.447831000
H	-6.096395000	-1.655989000	-0.802027000
C	-3.001042000	-2.316947000	-1.112598000
H	-3.289524000	-1.297194000	-1.374339000
C	-3.907680000	-3.348926000	-1.444066000
H	-4.653974000	-3.104317000	-2.204392000
H	-3.549961000	-4.377542000	-1.510561000
C	-1.726662000	-2.500040000	-0.540176000
C	-1.212601000	-3.868562000	-0.175423000
H	-2.009119000	-4.610202000	-0.050407000
H	-0.543379000	-4.233376000	-0.973846000
H	-0.642626000	-3.813784000	0.762812000
O	-0.913585000	-1.512556000	-0.363359000
C	-0.144478000	3.531009000	0.051533000
C	-1.469892000	3.074044000	-0.110935000
C	-2.541557000	3.956730000	-0.145770000
C	-2.321723000	5.324738000	-0.018655000
C	-1.005554000	5.768584000	0.141484000
C	0.089368000	4.907184000	0.180417000
C	0.846147000	2.469208000	0.064601000
H	-3.154234000	6.028436000	-0.044342000
H	1.092990000	5.314802000	0.307803000
C	0.447373000	1.150561000	-0.069409000
C	3.731567000	-1.334336000	0.007783000
C	3.960268000	0.052414000	0.136064000
C	5.276977000	0.514087000	0.270241000
C	6.308780000	-0.422362000	0.268478000
C	6.091092000	-1.797373000	0.138847000
C	4.780186000	-2.245387000	0.007771000
C	1.520556000	0.241479000	-0.045483000
C	2.751628000	0.855653000	0.103398000
H	5.507969000	1.575080000	0.371541000
H	6.923119000	-2.502178000	0.139325000
S	2.576484000	2.583985000	0.221059000
I	1.668607000	-1.839026000	-0.175249000
I	-1.619707000	0.954970000	-0.300617000
F	7.561016000	0.007552000	0.394464000
F	-0.793088000	7.076094000	0.261920000
F	-3.777458000	3.483969000	-0.301905000
F	4.521629000	-3.545249000	-0.119372000

TS-cat1-F8: E= -2867.13021689 (i = -331 cm^{-1})

C	-3.520040000	-2.868292000	1.793955000
C	-4.766885000	-2.330623000	1.158754000
C	-5.564569000	-3.365596000	0.324171000
C	-4.836679000	-4.678389000	0.103738000
C	-3.938307000	-5.130973000	1.071488000
C	-3.240830000	-4.203567000	1.890859000
H	-6.499457000	-3.619370000	0.849707000
H	-5.392977000	-1.950748000	1.986774000
H	-4.531231000	-1.429153000	0.572366000
H	-2.875030000	-2.149073000	2.309155000
H	-5.392027000	-5.435800000	-0.457027000
H	-3.640882000	-6.183258000	1.081127000
H	-2.402746000	-4.548264000	2.499939000

H	-5.872404000	-2.937468000	-0.641027000
C	-2.740735000	-3.088877000	-1.085724000
H	-3.210093000	-2.143753000	-1.366114000
C	-3.464954000	-4.270718000	-1.357435000
H	-4.274565000	-4.181905000	-2.086308000
H	-2.940358000	-5.225593000	-1.409706000
C	-1.442662000	-3.031585000	-0.546756000
C	-0.681979000	-4.275006000	-0.168198000
H	-1.324412000	-5.152265000	-0.036187000
H	0.044833000	-4.515539000	-0.963288000
H	-0.133836000	-4.106971000	0.769653000
O	-0.810434000	-1.908040000	-0.414888000
C	-0.964863000	3.156632000	0.026494000
C	-2.190820000	2.484926000	-0.162149000
C	-3.402197000	3.152640000	-0.201909000
C	-3.410581000	4.545958000	-0.050096000
C	-2.206693000	5.238516000	0.140027000
C	-0.993042000	4.547582000	0.177932000
C	0.205176000	2.305276000	0.040216000
C	0.046478000	0.938572000	-0.114417000
C	3.721133000	-0.930668000	-0.043479000
C	3.685104000	0.472619000	0.101901000
C	4.900798000	1.149266000	0.252488000
C	6.106516000	0.444020000	0.259011000
C	6.112616000	-0.950357000	0.115710000
C	4.905939000	-1.646786000	-0.037650000
C	1.263251000	0.236677000	-0.092772000
C	2.363342000	1.060543000	0.073019000
S	1.885697000	2.729662000	0.215060000
I	1.773063000	-1.782227000	-0.256335000
I	-1.948865000	0.370982000	-0.375665000
F	7.251273000	1.088557000	0.400929000
F	-2.227105000	6.552237000	0.283211000
F	-4.542549000	2.497240000	-0.380782000
F	4.915619000	-2.966506000	-0.173201000
F	4.911190000	2.471420000	0.388121000
F	7.260148000	-1.605099000	0.123722000
F	-4.552386000	5.210329000	-0.083455000
F	0.140244000	5.217834000	0.358125000

TS-cat1-NMe$_2$: E= -2341.02335295 (i = -393 cm^{-1})

C	-4.050438000	-2.043422000	1.911872000
C	-5.033716000	-0.911182000	1.884878000
C	-6.380650000	-1.257630000	1.194337000
C	-6.399724000	-2.630149000	0.540671000
C	-5.642008000	-3.660587000	1.112334000
C	-4.423219000	-3.355934000	1.761552000
H	-7.188528000	-1.258665000	1.943301000
H	-5.213271000	-0.639852000	2.941529000
H	-4.560898000	-0.018653000	1.448986000
H	-3.029576000	-1.806823000	2.226289000
H	-7.350341000	-2.910529000	0.077067000
H	-5.879565000	-4.701712000	0.876912000
H	-3.720087000	-4.158937000	1.992907000
H	-6.655070000	-0.486804000	0.458405000
C	-4.142215000	-1.669416000	-0.893669000
H	-4.218214000	-0.585512000	-0.799466000
C	-5.338418000	-2.370543000	-1.155006000
H	-6.151404000	-1.783567000	-1.590921000
H	-5.292234000	-3.390137000	-1.540754000
C	-2.841608000	-2.224135000	-0.784689000

C	-2.604988000	-3.710062000	-0.905310000
H	-3.524175000	-4.305069000	-0.899910000
H	-2.067903000	-3.909708000	-1.847493000
H	-1.974319000	-4.053074000	-0.072195000
O	-1.808511000	-1.483379000	-0.641817000
C	0.274097000	3.240303000	-0.053225000
C	-1.115428000	3.182425000	-0.290947000
C	-1.894973000	4.332291000	-0.339499000
C	-1.282015000	5.567073000	-0.146794000
C	0.120045000	5.682511000	0.099514000
C	0.882255000	4.483770000	0.140044000
C	0.941019000	1.950588000	-0.037128000
H	-2.969831000	4.291617000	-0.524914000
H	-1.908955000	6.455427000	-0.189381000
H	1.954756000	4.511077000	0.323797000
C	0.223255000	0.784112000	-0.231664000
C	2.791277000	-2.477738000	-0.130950000
C	3.335033000	-1.189879000	0.060216000
C	4.710702000	-1.055319000	0.267080000
C	5.560270000	-2.194474000	0.285623000
C	4.957053000	-3.474075000	0.088436000
C	3.587794000	-3.616945000	-0.117529000
C	1.026028000	-0.375435000	-0.202961000
C	2.366229000	-0.108956000	0.013216000
H	5.115820000	-0.055721000	0.413790000
H	5.564918000	-4.376558000	0.093845000
H	3.167596000	-4.613996000	-0.264151000
S	2.634482000	1.604422000	0.188090000
I	0.672502000	-2.422844000	-0.424194000
I	-1.809889000	1.170729000	-0.534054000
N	0.707352000	6.901610000	0.289826000
C	-0.091639000	8.116853000	0.244066000
H	-0.876465000	8.122391000	1.020668000
H	0.556910000	8.983372000	0.416324000
H	-0.579952000	8.249891000	-0.737114000
N	6.906441000	-2.069752000	0.484339000
C	7.758683000	-3.249029000	0.500206000
H	7.468533000	-3.953847000	1.298788000
H	7.728796000	-3.790509000	-0.461632000
H	8.795372000	-2.944041000	0.682975000
C	7.495889000	-0.753651000	0.671272000
H	7.093362000	-0.247729000	1.566537000
H	8.579473000	-0.854810000	0.800666000
H	7.319038000	-0.098373000	-0.199787000
C	2.136076000	6.988918000	0.545866000
H	2.420803000	6.451777000	1.467964000
H	2.727678000	6.572561000	-0.288390000
H	2.421078000	8.040152000	0.667531000

TS-cat1-NO_2: E= -2482.12833703 (i = -371 cm^{-1})

C	-4.440849000	-1.821560000	1.784201000
C	-5.401074000	-0.780668000	1.292079000
C	-6.510943000	-1.322234000	0.353963000
C	-6.315493000	-2.769035000	-0.066066000
C	-5.673422000	-3.649118000	0.811181000
C	-4.687121000	-3.166349000	1.708301000
H	-7.482948000	-1.280187000	0.871255000
H	-5.856228000	-0.334383000	2.195436000
H	-4.845274000	0.051900000	0.833754000
H	-3.570677000	-1.467491000	2.345925000
H	-7.110404000	-3.184380000	-0.692492000

H	-5.795069000	-4.727707000	0.678125000
H	-4.042179000	-3.875025000	2.232182000
H	-6.618607000	-0.683148000	-0.534919000
C	-3.797763000	-1.885450000	-1.096417000
H	-3.915850000	-0.807909000	-1.219194000
C	-4.886893000	-2.693684000	-1.494076000
H	-5.594696000	-2.232213000	-2.187983000
H	-4.725861000	-3.754562000	-1.691229000
C	-2.557989000	-2.348944000	-0.611555000
C	-2.282810000	-3.819477000	-0.426187000
H	-3.190053000	-4.432392000	-0.398955000
H	-1.660657000	-4.179972000	-1.263055000
H	-1.735006000	-3.979343000	0.513474000
O	-1.578455000	-1.544933000	-0.367299000
C	0.200420000	3.251052000	-0.023558000
C	-1.199908000	3.099604000	-0.142107000
C	-2.084592000	4.170323000	-0.173631000
C	-1.554265000	5.460419000	-0.080931000
C	-0.171079000	5.619850000	0.037679000
C	0.718193000	4.550549000	0.067421000
C	0.958138000	2.013283000	-0.009450000
H	-3.162897000	4.027675000	-0.265819000
H	-2.201044000	6.336708000	-0.099659000
H	1.788088000	4.736833000	0.159825000
C	0.299499000	0.800942000	-0.111327000
C	3.046846000	-2.306405000	-0.030646000
C	3.522589000	-0.978402000	0.061383000
C	4.906850000	-0.780281000	0.159622000
C	5.736415000	-1.896950000	0.160341000
C	5.255865000	-3.205723000	0.067079000
C	3.877872000	-3.419318000	-0.030893000
C	1.168594000	-0.305322000	-0.085463000
C	2.500107000	0.050689000	0.032375000
H	5.338808000	0.217597000	0.233196000
H	5.954414000	-4.041581000	0.070839000
H	3.486256000	-4.435182000	-0.103758000
S	2.678950000	1.780323000	0.120974000
I	0.905624000	-2.372985000	-0.172960000
I	-1.767508000	1.032062000	-0.281517000
N	7.201268000	-1.682485000	0.263628000
O	7.913689000	-2.670481000	0.285083000
O	7.598087000	-0.531180000	0.320238000
N	0.381102000	6.993506000	0.138525000
O	1.586925000	7.105566000	0.278908000
O	-0.405642000	7.921603000	0.075545000

TS-cat2': E= -2465.95775923 (i = -435 cm^{-1})

C	-2.929058000	-1.361594000	1.684437000
C	-3.794691000	-0.257026000	2.221044000
C	-5.264570000	-0.686277000	2.475800000
C	-5.578796000	-2.091723000	1.982368000
C	-4.574348000	-3.068558000	2.085097000
C	-3.230356000	-2.690509000	1.881658000
H	-5.466850000	-0.679667000	3.558817000
H	-3.329529000	0.059863000	3.172708000
H	-3.727475000	0.625727000	1.568408000
H	-1.934171000	-1.077889000	1.328918000
H	-6.609330000	-2.419050000	2.152513000
H	-4.843270000	-4.128460000	2.099012000
H	-2.470103000	-3.455928000	1.706164000
H	-5.966785000	0.035522000	2.032526000

C	-4.518168000	-1.237871000	-0.511081000
H	-4.432524000	-0.150856000	-0.478930000
C	-5.687124000	-1.841583000	-0.005340000
H	-6.565250000	-1.197259000	0.092091000
H	-5.933100000	-2.858550000	-0.316612000
C	-3.467251000	-1.931068000	-1.196912000
C	-3.571061000	-3.426509000	-1.391768000
H	-3.825934000	-3.943475000	-0.454729000
H	-4.374813000	-3.649611000	-2.112583000
H	-2.629033000	-3.828240000	-1.786058000
O	-2.471799000	-1.327467000	-1.681552000
H	1.702583000	3.144999000	-2.811502000
C	1.990294000	2.242116000	-2.270722000
C	2.603434000	-0.081783000	-0.879539000
C	1.516799000	2.059739000	-0.967818000
C	2.791494000	1.273176000	-2.869580000
C	3.077918000	0.096125000	-2.178928000
C	1.857777000	0.916161000	-0.222024000
H	3.161703000	1.417204000	-3.885577000
H	3.656671000	-0.702637000	-2.644667000
N	2.860948000	-1.344532000	-0.243607000
N	0.622798000	3.055684000	-0.455928000
C	-0.730623000	2.943020000	-0.421833000
N	-1.246716000	4.056302000	0.120328000
C	0.992231000	4.291167000	0.080806000
C	-0.203701000	4.930457000	0.443718000
C	2.040634000	-2.429515000	-0.299043000
N	2.635789000	-3.461403000	0.315888000
C	4.040043000	-1.703339000	0.419122000
C	3.888657000	-3.050125000	0.782230000
C	-2.657419000	4.362220000	0.342342000
H	-2.981860000	5.144452000	-0.358700000
H	-3.257493000	3.458197000	0.192112000
H	-2.784973000	4.714245000	1.374795000
C	2.114084000	-4.812673000	0.502567000
H	1.953117000	-4.995925000	1.573899000
H	1.167687000	-4.921088000	-0.037357000
H	2.843439000	-5.533386000	0.109459000
C	-0.206466000	6.204724000	1.018853000
C	2.241722000	4.880641000	0.281789000
C	5.192221000	-0.977235000	0.724517000
C	4.885211000	-3.738487000	1.480675000
H	-1.132366000	6.708236000	1.297568000
H	3.163816000	4.369390000	0.002881000
H	4.770135000	-4.783870000	1.767769000
H	5.301117000	0.068282000	0.436363000
C	1.038764000	6.799922000	1.217491000
C	2.239910000	6.149992000	0.858450000
C	6.188115000	-1.662710000	1.418062000
C	6.036810000	-3.016532000	1.790417000
H	1.086301000	7.795053000	1.663942000
H	3.191289000	6.655124000	1.036472000
H	6.843456000	-3.511037000	2.335078000
H	7.108980000	-1.138840000	1.681823000
C	1.501447000	0.721119000	1.247422000
I	-1.731328000	1.201817000	-1.071169000
I	0.131607000	-2.352218000	-1.154556000
F	2.611371000	0.442064000	1.949709000
F	0.650995000	-0.307773000	1.425643000
F	0.939953000	1.798522000	1.804297000

Adduct-1: E= -464.902924001 (i = 0)

C	0.272004000	-0.875921000	0.722358000
C	1.628968000	-1.451031000	0.233565000
C	2.392394000	-0.360064000	-0.564344000
C	1.559191000	0.950077000	-0.565529000
C	1.239858000	1.311781000	0.866102000
C	0.559986000	0.365733000	1.533255000
H	3.381522000	-0.167339000	-0.122083000
H	2.210288000	-1.780546000	1.108051000
H	1.444048000	-2.342885000	-0.386166000
H	-0.286718000	-1.633357000	1.289539000
H	2.103960000	1.752576000	-1.083896000
H	1.535795000	2.270878000	1.300214000
H	0.238039000	0.461366000	2.573701000
H	2.562755000	-0.678359000	-1.605667000
C	-0.543035000	-0.479863000	-0.565281000
H	-0.579504000	-1.382885000	-1.193763000
C	0.217951000	0.659651000	-1.295223000
H	0.422794000	0.366317000	-2.336883000
H	-0.374023000	1.584052000	-1.340392000
C	-1.999798000	-0.231358000	-0.176636000
C	-2.468961000	1.176369000	0.119356000
H	-1.734139000	1.718855000	0.733011000
H	-2.577440000	1.736234000	-0.824780000
H	-3.444531000	1.137652000	0.621619000
O	-2.756773000	-1.179350000	-0.072280000

Adduct-BF_3: E= -789.648539101 (i = 0)

C	-0.931067000	-0.439066000	-0.928112000
C	-1.602410000	-1.780671000	-0.530085000
C	-2.701506000	-1.511224000	0.531238000
C	-2.814428000	0.017472000	0.780550000
C	-2.997536000	0.712935000	-0.546709000
C	-2.006353000	0.492603000	-1.425108000
H	-3.673995000	-1.902719000	0.198038000
H	-2.028715000	-2.233003000	-1.437678000
H	-0.832724000	-2.471550000	-0.153312000
H	-0.130342000	-0.611672000	-1.655945000
H	-3.634046000	0.235769000	1.479582000
H	-3.860725000	1.352069000	-0.750540000
H	-1.967119000	0.925952000	-2.427686000
H	-2.462275000	-2.012048000	1.483065000
C	-0.306222000	0.122014000	0.428322000
H	0.276429000	-0.700757000	0.855002000
C	-1.469078000	0.505413000	1.383758000
H	-1.301549000	0.032839000	2.363382000
H	-1.523833000	1.588218000	1.558483000
C	0.663170000	1.204825000	0.084622000
C	0.229467000	2.618678000	-0.076501000
H	-0.761462000	2.668984000	-0.550306000
H	0.124130000	3.059853000	0.930271000
H	0.979670000	3.190027000	-0.636481000
O	1.872299000	0.959491000	-0.128727000
B	2.643714000	-0.436915000	0.021137000
F	2.518478000	-0.801762000	1.342583000
F	2.025088000	-1.318581000	-0.837953000
F	3.924809000	-0.132011000	-0.342808000

Adduct-cat1: E= -2073.00578966 (i = 0)

C	-4.197213000	-0.565122000	0.869414000
C	-5.326696000	0.438147000	0.507603000

C	-6.416354000	-0.290231000	-0.323070000
C	-6.020676000	-1.781672000	-0.492026000
C	-5.775978000	-2.366034000	0.878699000
C	-4.820761000	-1.739590000	1.583601000
H	-7.396319000	-0.223526000	0.171241000
H	-5.742454000	0.840124000	1.442957000
H	-4.903538000	1.289754000	-0.049342000
H	-3.419861000	-0.073710000	1.471763000
H	-6.799253000	-2.327460000	-1.042744000
H	-6.336222000	-3.228808000	1.248004000
H	-4.509708000	-2.027149000	2.590781000
H	-6.524766000	0.168025000	-1.318851000
C	-3.586809000	-1.053446000	-0.509253000
H	-3.310659000	-0.148920000	-1.071009000
C	-4.686138000	-1.832792000	-1.284507000
H	-4.825308000	-1.375984000	-2.275644000
H	-4.404747000	-2.879575000	-1.458906000
C	-2.293248000	-1.781483000	-0.242949000
C	-2.283844000	-3.265625000	-0.047593000
H	-3.205106000	-3.598670000	0.449490000
H	-2.255346000	-3.749030000	-1.039652000
H	-1.408331000	-3.586110000	0.530729000
O	-1.239859000	-1.133147000	-0.154998000
C	1.343684000	3.515230000	0.036588000
C	-0.063204000	3.579522000	-0.034514000
C	-0.786087000	4.762206000	-0.046884000
C	-0.056966000	5.957139000	0.014646000
C	1.342479000	5.938025000	0.086207000
C	2.043860000	4.732905000	0.097978000
C	1.908675000	2.180467000	0.038759000
H	-1.875078000	4.774508000	-0.103247000
H	-0.593433000	6.907961000	0.006300000
H	1.893691000	6.878932000	0.133738000
H	3.134497000	4.729252000	0.154646000
C	1.089037000	1.065538000	-0.020558000
C	3.451088000	-2.374552000	0.009514000
C	4.073410000	-1.110411000	0.068961000
C	5.477582000	-1.082290000	0.131758000
C	6.196125000	-2.277067000	0.131374000
C	5.538490000	-3.513083000	0.069160000
C	4.140054000	-3.577838000	0.007056000
C	1.806598000	-0.150664000	-0.009168000
C	3.178134000	0.029243000	0.055469000
H	5.997953000	-0.123285000	0.180440000
H	7.286174000	-2.246906000	0.179906000
H	6.113596000	-4.441089000	0.068522000
H	3.628387000	-4.540137000	-0.041402000
S	3.585726000	1.720248000	0.105505000
I	1.315213000	-2.188543000	-0.064265000
I	-0.931220000	1.620691000	-0.126932000

Adduct-cat1-F2: E= -2271.56197581 (i = 0)

C	-4.379496000	-0.803083000	0.932843000
C	-5.548617000	0.176302000	0.637733000
C	-6.646746000	-0.560107000	-0.174153000
C	-6.215941000	-2.034366000	-0.398409000
C	-5.907988000	-2.647097000	0.947054000
C	-4.946159000	-2.012781000	1.635657000
H	-7.609536000	-0.535009000	0.356517000
H	-5.941289000	0.540983000	1.597977000
H	-5.168308000	1.053679000	0.091045000

H	-3.595618000	-0.304730000	1.520760000
H	-6.997890000	-2.587408000	-0.936933000
H	-6.432080000	-3.533973000	1.312249000
H	-4.592912000	-2.317306000	2.623774000
H	-6.804934000	-0.077976000	-1.151819000
C	-3.804254000	-1.237987000	-0.479350000
H	-3.572098000	-0.312816000	-1.027901000
C	-4.909377000	-2.026484000	-1.237496000
H	-5.095536000	-1.546489000	-2.209598000
H	-4.606190000	-3.059667000	-1.451815000
C	-2.486440000	-1.940738000	-0.273161000
C	-2.439383000	-3.426585000	-0.097763000
H	-3.343328000	-3.788941000	0.409733000
H	-2.414497000	-3.895111000	-1.097011000
H	-1.547393000	-3.734909000	0.461483000
O	-1.443094000	-1.271283000	-0.215645000
C	1.036145000	3.419317000	0.024488000
C	-0.371355000	3.451381000	-0.069815000
C	-1.113780000	4.624284000	-0.087231000
C	-0.418771000	5.834083000	-0.009565000
C	0.976600000	5.820586000	0.082560000
C	1.718114000	4.643217000	0.102531000
C	1.629434000	2.096469000	0.029784000
H	-0.946704000	6.788682000	-0.020475000
H	2.805653000	4.690774000	0.176653000
C	0.831517000	0.966980000	-0.051204000
C	3.253137000	-2.424699000	-0.017701000
C	3.853576000	-1.151271000	0.079670000
C	5.251499000	-1.088428000	0.184095000
C	5.973391000	-2.277780000	0.180679000
C	5.363074000	-3.531829000	0.077739000
C	3.971400000	-3.613368000	-0.022488000
C	1.571999000	-0.234317000	-0.033998000
C	2.938310000	-0.027057000	0.058942000
H	5.778957000	-0.136919000	0.268030000
H	5.980575000	-4.431199000	0.078419000
S	3.312554000	1.669876000	0.125293000
I	1.120844000	-2.278109000	-0.133712000
I	-1.196780000	1.481112000	-0.187957000
F	7.299940000	-2.217730000	0.279572000
F	1.623251000	6.982218000	0.154078000
H	-2.201736000	4.620113000	-0.162024000
H	3.485337000	-4.586923000	-0.101592000

Adduct-cat1-F4: E= -2470.11059602 (i = 0)

C	-4.242439000	-1.734278000	1.015845000
C	-5.540849000	-0.944505000	0.692586000
C	-6.405435000	-1.763853000	-0.302216000
C	-5.717691000	-3.127748000	-0.581313000
C	-5.428342000	-3.797947000	0.739887000
C	-4.644913000	-3.081936000	1.561630000
H	-7.412187000	-1.936713000	0.104596000
H	-6.078779000	-0.768410000	1.635258000
H	-5.281646000	0.043952000	0.280324000
H	-3.609759000	-1.168376000	1.714332000
H	-6.344472000	-3.750193000	-1.234730000
H	-5.821885000	-4.787677000	0.984794000
H	-4.327507000	-3.413814000	2.552925000
H	-6.531968000	-1.223723000	-1.253988000
C	-3.501344000	-1.910202000	-0.376477000
H	-3.457662000	-0.913406000	-0.836533000

C	-4.358544000	-2.846729000	-1.277321000
H	-4.525518000	-2.360123000	-2.249579000
H	-3.849135000	-3.798155000	-1.482831000
C	-2.077552000	-2.336407000	-0.152254000
C	-1.748382000	-3.773946000	0.105718000
H	-2.605183000	-4.313515000	0.528701000
H	-1.499518000	-4.250294000	-0.858970000
H	-0.881763000	-3.862965000	0.774646000
O	-1.170880000	-1.484812000	-0.156264000
C	0.372895000	3.572165000	0.040974000
C	-1.008277000	3.309025000	-0.060869000
C	-1.953808000	4.326478000	-0.076526000
C	-1.539163000	5.651511000	0.010977000
C	-0.168519000	5.909599000	0.115117000
C	0.797357000	4.904828000	0.132631000
C	1.206150000	2.384415000	0.040328000
H	-2.266514000	6.463953000	0.000114000
H	1.851724000	5.170314000	0.218832000
C	0.630994000	1.126192000	-0.040139000
C	3.603493000	-1.747979000	-0.018251000
C	3.993462000	-0.395021000	0.058741000
C	5.360426000	-0.092777000	0.134430000
C	6.272203000	-1.147180000	0.130540000
C	5.888790000	-2.489588000	0.054028000
C	4.530925000	-2.782113000	-0.021028000
C	1.580775000	0.083739000	-0.032479000
C	2.886100000	0.542787000	0.047383000
H	5.724328000	0.933432000	0.195987000
H	6.630663000	-3.288865000	0.053202000
S	2.938435000	2.278601000	0.120144000
I	1.497910000	-2.013012000	-0.103959000
I	-1.461476000	1.239891000	-0.173029000
F	7.569073000	-0.867547000	0.203211000
F	0.228091000	7.174479000	0.203036000
F	-3.244353000	4.021881000	-0.174325000
F	4.111408000	-4.041069000	-0.095318000

Adduct-cat1-F8: E= -2867.17478539 (i = 0)

C	-4.162707000	-2.657628000	0.914901000
C	-5.560115000	-2.103507000	0.520606000
C	-6.219245000	-3.059522000	-0.508711000
C	-5.293367000	-4.284158000	-0.741118000
C	-4.959444000	-4.894910000	0.598214000
C	-4.353465000	-4.054714000	1.451807000
H	-7.200623000	-3.402831000	-0.151157000
H	-6.165922000	-2.024604000	1.434801000
H	-5.457918000	-1.084588000	0.113681000
H	-3.672463000	-1.991493000	1.638894000
H	-5.769421000	-5.005359000	-1.419571000
H	-5.188082000	-5.937843000	0.831591000
H	-4.033003000	-4.326506000	2.460269000
H	-6.387437000	-2.548498000	-1.470040000
C	-3.330388000	-2.705253000	-0.436874000
H	-3.428573000	-1.714881000	-0.902655000
C	-3.970307000	-3.772752000	-1.372176000
H	-4.171686000	-3.318222000	-2.353335000
H	-3.297099000	-4.622353000	-1.549323000
C	-1.871528000	-2.884433000	-0.129746000
C	-1.313874000	-4.248794000	0.122108000
H	-2.086025000	-4.926124000	0.509230000
H	-0.963248000	-4.665408000	-0.838312000

H	-0.465930000	-4.207750000	0.818230000
O	-1.128741000	-1.886501000	-0.055904000
C	-0.452518000	3.326701000	0.001398000
C	-1.779917000	2.851021000	-0.006957000
C	-2.879702000	3.693400000	-0.005002000
C	-2.659012000	5.077968000	0.008024000
C	-1.351184000	5.582357000	0.017037000
C	-0.257905000	4.712671000	0.013432000
C	0.574105000	2.308213000	-0.002238000
C	0.209539000	0.970884000	-0.012747000
C	3.610257000	-1.389338000	-0.001868000
C	3.761985000	0.012626000	0.002490000
C	5.064045000	0.526142000	0.006537000
C	6.165403000	-0.333580000	0.005686000
C	5.980905000	-1.723292000	0.001141000
C	4.686269000	-2.261466000	-0.002293000
C	1.316647000	0.100617000	-0.008579000
C	2.530208000	0.770889000	0.000392000
S	2.303703000	2.495911000	0.008535000
I	1.569053000	-1.982868000	-0.002348000
I	-1.876615000	0.727356000	-0.034865000
F	7.389502000	0.160671000	0.008854000
F	-1.157386000	6.888085000	0.029107000
F	-4.116619000	3.218421000	-0.014971000
F	4.513106000	-3.575355000	-0.006060000
F	5.256539000	1.840023000	0.010847000
F	7.029549000	-2.525107000	-0.000080000
F	-3.683437000	5.910363000	0.011727000
F	0.974388000	5.206809000	0.021448000

Adduct-cat1-NMe$_2$: E= -2341.07499921 (i = 0)

C	-4.839582000	-1.292458000	0.942548000
C	-6.033907000	-0.351694000	0.624259000
C	-7.090323000	-1.124767000	-0.209063000
C	-6.608853000	-2.585627000	-0.419634000
C	-6.307538000	-3.185296000	0.933086000
C	-5.379525000	-2.518935000	1.637668000
H	-8.065021000	-1.128813000	0.300134000
H	-6.458260000	0.000988000	1.575542000
H	-5.670952000	0.537339000	0.083717000
H	-4.082855000	-0.768866000	1.543998000
H	-7.362561000	-3.164370000	-0.971427000
H	-6.809137000	-4.088471000	1.290003000
H	-5.034820000	-2.810489000	2.632743000
H	-7.241960000	-0.650294000	-1.191553000
C	-4.225273000	-1.708199000	-0.457400000
H	-4.020126000	-0.775326000	-1.002949000
C	-5.286187000	-2.539468000	-1.232093000
H	-5.468483000	-2.074394000	-2.212330000
H	-4.943479000	-3.563779000	-1.429761000
C	-2.880322000	-2.354933000	-0.233199000
C	-2.772339000	-3.837688000	-0.046270000
H	-3.666467000	-4.236968000	0.451060000
H	-2.711754000	-4.311068000	-1.041666000
H	-1.874087000	-4.100116000	0.526366000
O	-1.869246000	-1.642327000	-0.173116000
C	0.415774000	3.185444000	-0.008280000
C	-0.991941000	3.144209000	-0.081647000
C	-1.766729000	4.297435000	-0.100548000
C	-1.122225000	5.529476000	-0.046586000
C	0.300625000	5.635396000	0.028306000

C	1.051989000	4.428444000	0.046985000
C	1.067623000	1.888391000	-0.000330000
H	-2.855642000	4.261750000	-0.158954000
H	-1.741682000	6.423743000	-0.063926000
H	2.138694000	4.449679000	0.105902000
C	0.326324000	0.720565000	-0.059083000
C	2.905042000	-2.552538000	-0.047683000
C	3.453485000	-1.255143000	0.018384000
C	4.842160000	-1.112454000	0.081173000
C	5.694226000	-2.250589000	0.080656000
C	5.079957000	-3.538999000	0.011529000
C	3.698354000	-3.693328000	-0.051288000
C	1.124314000	-0.445470000	-0.051600000
C	2.481040000	-0.177181000	0.010880000
H	5.255105000	-0.106623000	0.131695000
H	5.688796000	-4.440845000	0.006450000
H	3.269218000	-4.695946000	-0.101237000
S	2.771854000	1.538373000	0.066040000
I	0.771828000	-2.511750000	-0.118373000
I	-1.726650000	1.139816000	-0.165955000
N	7.051228000	-2.117279000	0.144777000
C	7.905683000	-3.295723000	0.146446000
H	7.696248000	-3.951894000	1.009024000
H	7.784634000	-3.890816000	-0.775359000
H	8.953836000	-2.982364000	0.209427000
N	0.916402000	6.852452000	0.079390000
C	2.367393000	6.933037000	0.145998000
H	2.763456000	6.441473000	1.051948000
H	2.844160000	6.465779000	-0.733667000
H	2.672496000	7.985159000	0.173833000
C	7.651712000	-0.793972000	0.210345000
H	7.324479000	-0.239785000	1.107581000
H	8.742370000	-0.891304000	0.254627000
H	7.402143000	-0.186563000	-0.677463000
C	0.128431000	8.076290000	0.056641000
H	-0.464374000	8.165208000	-0.870413000
H	-0.564416000	8.133370000	0.914072000
H	0.800589000	8.940173000	0.109880000

Adduct-cat1-NO_2: E= -2482.17579020 (i = 0)

C	-4.747508000	-1.280428000	0.956879000
C	-5.957719000	-0.370442000	0.609369000
C	-6.982568000	-1.176139000	-0.232246000
C	-6.466977000	-2.628823000	-0.416343000
C	-6.173356000	-3.204962000	0.947975000
C	-5.270174000	-2.510657000	1.657790000
H	-7.965495000	-1.194856000	0.260207000
H	-6.404093000	-0.019147000	1.550876000
H	-5.607506000	0.521790000	0.065541000
H	-4.011438000	-0.733377000	1.563095000
H	-7.199050000	-3.230617000	-0.972286000
H	-6.660711000	-4.114216000	1.308896000
H	-4.933795000	-2.782423000	2.661164000
H	-7.126897000	-0.716846000	-1.222830000
C	-4.105562000	-1.693676000	-0.433970000
H	-3.924799000	-0.761960000	-0.989308000
C	-5.133749000	-2.565472000	-1.210191000
H	-5.310221000	-2.121327000	-2.201031000
H	-4.761737000	-3.584048000	-1.383521000
C	-2.751033000	-2.302767000	-0.192740000
C	-2.608949000	-3.771064000	0.062324000

H	-3.523141000	-4.189879000	0.501702000
H	-2.442916000	-4.274730000	-0.906532000
H	-1.751692000	-3.976304000	0.717267000
O	-1.746783000	-1.570123000	-0.177492000
C	0.458789000	3.230614000	0.001599000
C	-0.949256000	3.191919000	-0.100036000
C	-1.754886000	4.321809000	-0.135670000
C	-1.124354000	5.567775000	-0.065666000
C	0.267845000	5.622048000	0.036571000
C	1.073062000	4.488908000	0.071929000
C	1.123575000	1.942174000	0.017382000
H	-2.840562000	4.263585000	-0.218092000
H	-1.703988000	6.489827000	-0.090353000
H	2.154750000	4.593472000	0.153658000
C	0.384574000	0.773473000	-0.058406000
C	2.973870000	-2.489214000	-0.057681000
C	3.511007000	-1.186157000	0.033240000
C	4.905760000	-1.059719000	0.108016000
C	5.676751000	-2.217065000	0.088268000
C	5.130263000	-3.499787000	-0.001771000
C	3.741856000	-3.645549000	-0.076281000
C	1.186969000	-0.386197000	-0.047800000
C	2.541249000	-0.107264000	0.032225000
H	5.390710000	-0.086082000	0.179540000
H	5.786247000	-4.369395000	-0.012939000
H	3.297886000	-4.639922000	-0.145845000
S	2.826763000	1.605944000	0.105021000
I	0.835525000	-2.448316000	-0.142226000
I	-1.667907000	1.175345000	-0.196488000
N	7.153245000	-2.076966000	0.166724000
O	7.814473000	-3.099597000	0.161930000
O	7.606823000	-0.947627000	0.230324000
N	0.925329000	6.951739000	0.110864000
O	2.139724000	6.971886000	0.211298000
O	0.208214000	7.934877000	0.066510000

Adduct-cat2': E= -2466.01501144 (i = 0)

C	1.831774000	-2.366238000	1.207085000
C	1.186283000	-2.484098000	2.611029000
C	1.891681000	-3.616287000	3.403297000
C	3.031315000	-4.217807000	2.535121000
C	3.935998000	-3.093727000	2.087865000
C	3.312530000	-2.135002000	1.384920000
H	2.309920000	-3.235079000	4.346619000
H	1.284553000	-1.514390000	3.121097000
H	0.107326000	-2.678619000	2.499371000
H	1.349164000	-1.567144000	0.635028000
H	3.576384000	-4.989781000	3.096365000
H	5.004804000	-3.084652000	2.317697000
H	3.807943000	-1.252722000	0.972188000
H	1.181329000	-4.416496000	3.666027000
C	1.605093000	-3.752780000	0.487227000
H	0.524661000	-3.954351000	0.541360000
C	2.387757000	-4.842673000	1.266197000
H	1.702833000	-5.652200000	1.561363000
H	3.173699000	-5.302634000	0.653064000
C	1.904014000	-3.570503000	-0.989480000
C	3.231419000	-3.983214000	-1.558246000
H	4.048741000	-3.646362000	-0.902195000
H	3.285903000	-5.083870000	-1.599065000
H	3.346445000	-3.578505000	-2.571816000

O	1.074936000	-3.022508000	-1.714402000
H	-3.433520000	1.448406000	-2.723541000
C	-2.505113000	1.744679000	-2.233400000
C	-0.128155000	2.406171000	-0.966943000
C	-2.231116000	1.245767000	-0.955538000
C	-1.600525000	2.590252000	-2.869861000
C	-0.398369000	2.908971000	-2.239409000
C	-1.048751000	1.591937000	-0.276824000
H	-1.818844000	2.979741000	-3.865138000
H	0.345324000	3.537767000	-2.731186000
N	1.146765000	2.713663000	-0.381881000
N	-3.191396000	0.342314000	-0.394525000
C	-3.081370000	-1.011194000	-0.374622000
N	-4.176962000	-1.529317000	0.199949000
C	-4.404515000	0.710517000	0.194747000
C	-5.032060000	-0.487296000	0.570621000
C	2.287356000	2.000458000	-0.569033000
N	3.293137000	2.581627000	0.099791000
C	1.435738000	3.813575000	0.433563000
C	2.800811000	3.721028000	0.744315000
C	-4.478390000	-2.937545000	0.442878000
H	-5.418484000	-3.197111000	-0.062121000
H	-3.668754000	-3.562043000	0.051632000
H	-4.581283000	-3.102344000	1.524218000
C	4.682976000	2.139592000	0.201527000
H	4.879135000	1.795495000	1.226516000
H	4.864451000	1.322557000	-0.504444000
H	5.342484000	2.982575000	-0.041893000
C	-6.280066000	-0.496042000	1.200108000
C	-4.985120000	1.958420000	0.431116000
C	0.633101000	4.851421000	0.911102000
C	3.433715000	4.664840000	1.559581000
H	-6.770162000	-1.425241000	1.491452000
H	-4.488786000	2.884341000	0.138774000
H	4.493083000	4.593786000	1.806119000
H	-0.427379000	4.912011000	0.666557000
C	-6.864632000	0.747470000	1.436741000
C	-6.229000000	1.951161000	1.061093000
C	1.263035000	5.793691000	1.720898000
C	2.636437000	5.701541000	2.039861000
H	-7.838925000	0.791966000	1.927287000
H	-6.724638000	2.901326000	1.269642000
H	3.084918000	6.463505000	2.680148000
H	0.679717000	6.625180000	2.121113000
C	-0.740046000	1.190573000	1.162855000
I	-1.357046000	-2.024251000	-1.028159000
I	2.387044000	0.288088000	-1.739074000
F	-0.688083000	2.282747000	1.940421000
F	0.458993000	0.580231000	1.254726000
F	-1.639255000	0.361201000	1.698261000
